# Rapid antibiotic-resistance predictions from genome sequence data for *Staphylococcus aureus* and *Mycobacterium tuberculosis*

**DOI:** 10.1038/ncomms10063

**Published:** 2015-12-21

**Authors:** Phelim Bradley, N. Claire Gordon, Timothy M. Walker, Laura Dunn, Simon Heys, Bill Huang, Sarah Earle, Louise J. Pankhurst, Luke Anson, Mariateresa de Cesare, Paolo Piazza, Antonina A. Votintseva, Tanya Golubchik, Daniel J. Wilson, David H. Wyllie, Roland Diel, Stefan Niemann, Silke Feuerriegel, Thomas A. Kohl, Nazir Ismail, Shaheed V. Omar, E. Grace Smith, David Buck, Gil McVean, A. Sarah Walker, Tim E. A. Peto, Derrick W. Crook, Zamin Iqbal

**Affiliations:** 1 Wellcome Trust Centre for Human Genetics, University of Oxford, Oxford OX3 7BN, UK; 2 Nuffield Department of Medicine, University of Oxford, Oxford OX1 1NF, UK; 3 Institute for Epidemiology, University Medical Hospital Schleswig-Holstein, Niemannsweg 11, 24105 Kiel, Germany; 4 Molecular and Experimental Mycobacteriology, Research Centre Borstel, Parkallee 1, 23845 Borstel, Germany; 5 German Centre for Infection Research, Partner Site Borstel, Parkallee 1, 23845 Borstel, Germany; 6 Centre for Tuberculosis, National Institute for Communicable Diseases, Private Bag X4 Sandringham, Johannesburg 2131, South Africa; 7 Department of Medical Microbiology, University of Pretoria, PO Box 667, Pretoria 0001, South Africa; 8 Regional Centre for Mycobacteriology, PHE Public Health Laboratory Birmingham. Heartlands Hospital, Bordesley Green East, Birmingham B9 5SS, UK; 9 Biomedical Research Centre, NIHR (National Institutes of Health Research) Oxford Biomedical Research Centre, Oxford OX3 7LE, UK; 10 National Infection Service, Public Health England, Wellington House, 133-155 Waterloo Road, London SE1 8UG, UK

## Abstract

The rise of antibiotic-resistant bacteria has led to an urgent need for rapid detection of drug resistance in clinical samples, and improvements in global surveillance. Here we show how de Bruijn graph representation of bacterial diversity can be used to identify species and resistance profiles of clinical isolates. We implement this method for *Staphylococcus aureus* and *Mycobacterium tuberculosis* in a software package (‘Mykrobe predictor') that takes raw sequence data as input, and generates a clinician-friendly report within 3 minutes on a laptop. For *S*. *aureus*, the error rates of our method are comparable to gold-standard phenotypic methods, with sensitivity/specificity of 99.1%/99.6% across 12 antibiotics (using an independent validation set, *n*=470). For *M*. *tuberculosis*, our method predicts resistance with sensitivity/specificity of 82.6%/98.5% (independent validation set, *n*=1,609); sensitivity is lower here, probably because of limited understanding of the underlying genetic mechanisms. We give evidence that minor alleles improve detection of extremely drug-resistant strains, and demonstrate feasibility of the use of emerging single-molecule nanopore sequencing techniques for these purposes.

## Abstract (web summary)

The clinical application of new sequencing techniques is expected to accelerate pathogen identification. Here, Bradley *et al*. present a clinician-friendly software package that uses sequencing data for quick and accurate prediction of antibiotic resistance profiles for *S. aureus* and *M. tuberculosis*.

## Graphical abstract

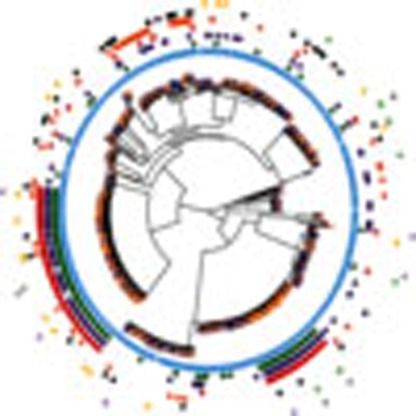

The marked increase in antibiotic use in health care and agriculture since the 1940s has driven a rise in frequency of drug-resistant bacterial strains, which now present a global threat to public health. Clinical isolates resistant to most drugs have now been seen for many species including *Mycobacterium tuberculosis*, *Enterococcus faecium*, *Staphylococcus aureus*, *Klebsiella pneumoniae*, *Neisseria gonorrhoeae*, *Acinetobacter baumannii* and *Pseudomonas aeruginosa*^1^. Antimicrobial susceptibility testing is therefore now central to the treatment of serious bacterial infections diagnosed by culture, and is used to determine the protocols for first-line antibiotic use when culture is not available. At present, phenotyping tests take at least 1–2 days to complete for rapidly growing bacteria such as *S*. *aureus*, and can take weeks in slow-growing bacteria such as *M*. *tuberculosis.*

Microbial genome sequencing has the potential to substantially increase the speed of antibiotic resistance detection for many pathogens^2^ and in addition provides valuable information on relatedness that could contribute to surveillance. The key biological constraint is the extent of our understanding of the genotype-to-phenotype correspondence—that is, the genotype needs to be sufficiently predictive of resistance. Increasingly, this correspondence is high for many bacterium/drug combinations. For example, Gordon *et al*.^3^ recently demonstrated that a curated panel of mutations and genes known to cause drug resistance in *S*. *aureus* was sufficient to predict resistance for 12 antibiotics with a sensitivity of 97% and specificity of 99%. Thus, at least for one species, a genotype-based method could deliver results with accuracy suitable for use in a clinical laboratory.

We set out to develop methods applicable to standard clinical samples, and solve the multiple computational challenges that act as barrier to routine and rapid deployment of such a system in clinical practice. These challenges include not only the need to determine species and predict resistance, but also developing a framework extensible to many species, and ensuring accessibility of the tool to a user base who may be unskilled in bioinformatics. Furthermore, although clinical samples currently undergo protocols that tend to remove diversity (for example, blood culture for *S*. *aureus* and solid/liquid culture for *M*. *tuberculosis*), future developments to reduce bed-to-diagnosis time will surely involve reductions in culture time, and potentially increase the levels of diversity in the sample. Thus we set out to build a system robust to mixture, and to establish whether an appreciable proportion of phenotypic resistance was explained by low-frequency alleles.

Methods using genome sequence data for species identification range from the specific^4^ to the sensitive^5^, but generally performance is measured globally in terms of detection of species presence. However, for clinical use we need considerable flexibility in tuning sensitivity and specificity for different species, potentially weighted to minimize clinical risk. For example, there may be a species associated with high mortality (for example, *S*. *aureus*) that can occur in samples mixed with other species (for example, coagulase-negative staphylococci (CoNS), which are common contaminants of blood cultures, being present on the skin through which the blood was taken), and that may even share the same resistance genes and thus confound inference.

Various methods have been used for genotyping resistance features: mutations and genes have been detected by whole-genome assembly^3^, genes by assembly and BLAST^6^, or single nucleotide polymorphisms (SNPs) and indels by mapping^7^,^8^. These methods have been demonstrated to have adequate performance in some circumstances. However, traditional whole-genome bacterial assembly is fundamentally based on the assumption that all data comes from a single haploid genome^9^, and so is ill-suited for mixed samples, and mapping to a single-reference results in error rates that depend on genetic distance of the sample from the reference^10^. There are pre-existing tools for expert users that incorporate resistance prediction^6^,^11^,^12^, none of which handle the issue of contaminating related species in clinical samples, or minor clones—we include comparative data below.

Here we aim to move beyond proof-of-concept of how sequencing might work in the clinic, to a general framework for genotype-based antimicrobial-resistance prediction, with concrete implementations for two species where drug resistance is of global concern: *S*. *aureus* and *M*. *tuberculosis*. The user should be able to obtain a report interpretable by a clinician by simple drag-and-drop of raw sequence data file. We evaluate extensively against clinical gold standards using current (Illumina) sequence data, including the impact of minor population detection. We notice that minor alleles improve ability to distinguish multi-drug resistant (MDR) from extensively drug-resistant (XDR) tuberculosis. We demonstrate that our method also works with an emerging strand-sequencing technology (Oxford Nanopore Technologies (ONT), MinION), giving perfect concordance with phenotype after just 7 h of sequencing, both for SNP-based and gene-based resistance. Finally, we discuss what is needed to apply our framework to other species.

## Results

### Using population genome graphs for genotyping

**Figure 1:**
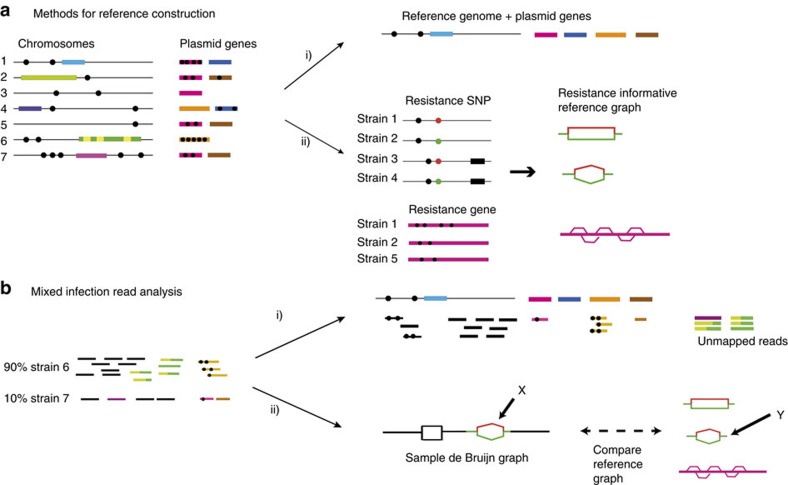
Representation and analysis of bacterial genetic variation. (**a**) Reference construction methods. Left: chromosomes with SNPs (black circles) and genes (coloured blocks) from strains of a bacterial species. Option (i) picks strain 1 to be reference, plus one example of each plasmid resistance gene. In option (ii), our method is to build the de Bruijn graph of all strains, restrict to loci of interest, and annotate resistance (red) and susceptible (green) alleles. For SNPs, local graph topology is determined by adjacent SNPs (black dots) and indels (black blocks). (**b**) Mixed infection read analysis. Left: sequence data from a clinical sample harbouring major (90%) and minor (10%) strains. Right: option (i) maps the reads to the reference genome to detect SNPs and genes. In option (ii), our approach, we construct the de Bruijn graph of the sample and compare with the reference graph. We see a specific SNP is present both in the sample and the reference graph (marked X, Y). Both the resistant (red) and susceptible (green) alleles are present in the sample, and within-sample frequency is estimated from sequencing depth on each allele.

We show in Fig. 1a a cartoon of the genetic diversity in a bacterial species and two options for building a reference variation structure. In option (i) we show the standard approach, where we select an arbitrary strain (strain 1) to be the reference genome, along with one copy of each plasmid gene. In this work we have developed an alternate approach, shown in option (ii). We start with a curated knowledge base of resistant/susceptible alleles, and assemble a de Bruijn graph^13^ of them on different genetic backgrounds, along with many examples of resistance genes. This forms our reference graph. In Fig. 1b we show the corresponding analyses of a mixed sample. The traditional approach is shown in option (i), whereby sequence reads are mapped to the reference genome and genes, requiring the mapping and inference to cope with the divergence between sample strains and the reference. Our approach (option (ii)) directly compares the de Bruijn graph of the sample with the reference graph. This results in statistical tests for the presence of resistance alleles that are unbiased by choice of reference or assumptions of clonality. Moreover, these tests will improve as the catalogue of diversity in the species grows. Our approach is implemented in a software application called Mykrobe predictor. See Methods section for details.

### *S*. *aureus* species identification

**Figure 2:**
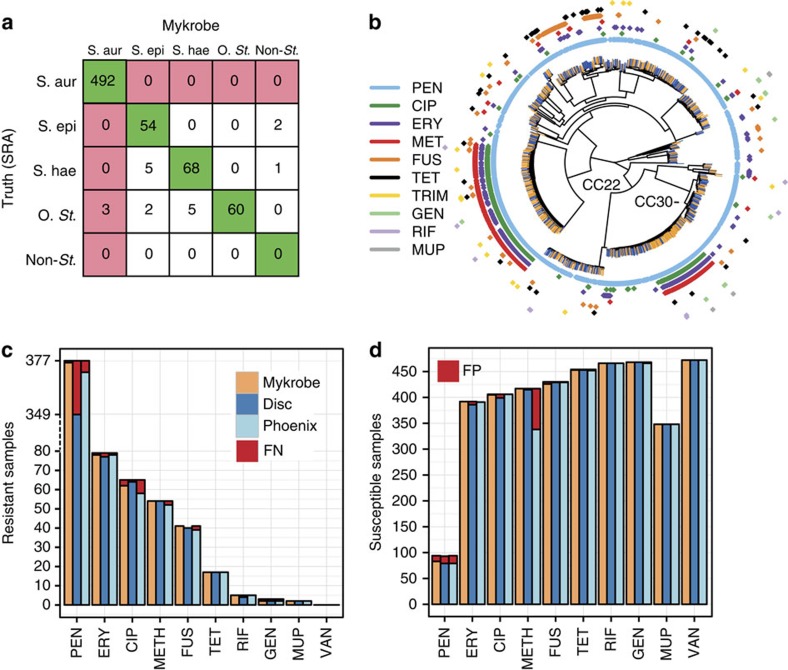
Species and susceptibility predictions for *S*. *aureus*. (**a**) Species classification results on species validation set St_B (*n*=692). Red shading of box indicates errors we wish to minimize. S.aur, *S*. *aureus*; S.epi, *S*. *epidermidis*; S.hae, *S*. *haemolyticus*; O.st, other staphylococcus; Non-st, non staphylococcal. ‘Truth (SRA)' is the species as annotated in the SRA metadata, which was used as truth for comparisons. (**b**) Phylogeny of *S*. *aureus* samples used in evaluating resistance prediction, with tips marked orange or blue to represent samples in training set (St_A1, *n*=495) or validation set (St_B1, *n*=471). Drug resistance is indicated in concentric rings around the phylogenetic tree; plasmid-mediated resistance (erythromycin in purple, tetracycline in black) is distributed across the whole tree. The two multi-drug resistant clades are in UK hospital clonal complexes CC22 and CC30. (**c**) Proportion of resistant *S*. *aureus* samples (St_B1) correctly identified as resistant by Mykrobe predictor (orange), disc test (dark blue) and Phoenix (light blue) compared with consensus, with false negatives in red. Note the break in the *y* axis between 80 and over 300 to show penicillin on same plot. (**d**) As **c**, but showing proportion of susceptible samples correctly identified as susceptible—false positives in red. A small number of failed disc tests for fusidic acid in panel **c** result in a lower bar. PEN, penicillin; ERY, erythromycin; CIP, ciprofloxacin; METH, methicillin; FUS, fusidic acid; CLIN, clindamycin; TET, tetracycline; RIF, rifampicin; GEN, gentamicin; MUP, mupirocin; TRIM, trimethoprim; VAN, vancomycin.

All the data sets in this study are Illumina sequence data sets, and are described in Methods section, Supplementary Fig. 1 and Supplementary Table 1. We designed several panels of probes for detection of *S*. *aureus*, *S*. *epidermidis*, *S*. *haemolyticus* or other CoNS (see Methods section for details). We then evaluated our predictions on a separate validation set (St_B), combining 471 *S*. *aureus* samples (St_B1, Supplementary Data 3), and 221 CoNS (St_B2, Supplementary Data 4) and show the results in Fig. 2a. This confirmed an appropriately low rate of missing a true *S*. *aureus* sample (0/492, upper 97.5% confidence interval 0.7%). We studied the three non-*S*. *aureus* samples that appeared to be misclassified by Mykrobe predictor as *S*. *aureus*, and concluded that they were mis-labelled in the National Center for Biotechnology Information's Short Read Archive (SRA), as both BLAST and OneCodex ( http://beta.onecodex.com) agreed with Mykrobe predictor that these were *S*. *aureus*. We also created 540 *in silico* mixtures of *S*. *epidermidis*/*S*. *aureus* and *S*. *haemolyticus/S*. *aureus* (simulation 1) and correctly detected presence of *S*. *epidermidis and S*. *haemolyticus* minor infections in 100% of cases at frequencies above 0.7% (see Supplementary Fig. 2 and Methods section).

### Comparing *S. aureus* predictions with consensus phenotype

We used a training set (St_A1) of 495 and a validation set (St_B1) of 471 *S*. *aureus* isolates that had been sequenced and phenotyped after being collected in Oxfordshire, UK (samples and phenotyping described in Methods section, Supplementary Fig. 1; accession codes and phenotyping data are given in Supplementary Data 1 and 3). We show in Fig. 2b a phylogeny (construction described in Methods section) of these samples, showing that both the training set (orange tips of tree) and validation set (blue) are distributed across the entire phylogeny. We also confirmed that, although enriched for the *S*. *aureus* clonal complexes seen in UK hospitals (CC22/CC30), all major clonal complexes were represented (Supplementary Fig. 3). A subset of these samples (*n*=94) has been compared in a previous publication with international clinical strains (US, UK, Japan and Australia), animal-associated strains (bovine, ovine and poultry) and historic clinical strains (1943, 1952 and 1960) to show that Oxfordshire *S*. *aureus* diversity, while not exhaustive, broadly recapitulates that of the world^14^.

All validation samples were phenotyped using two methods: a British Society for Antimicrobial Chemotherapy (BSAC) disc test^15^ and the Phoenix automated microbiology system (BD Biosciences, Sparks, MD, USA), except for trimethoprim, for which only disc testing was performed. A ‘consensus' phenotype was defined to be either that called by disc/Phoenix where they agreed, or the result of an Etest and/or nitrocefin (for penicillin) where disc and Phoenix were discrepant. This allowed us to estimate error rates for disc and Phoenix as well as for Mykrobe predictor.

Considering each resistance mutation and gene in turn, our prediction algorithm first genotypes a sample into one of three categories: clonal susceptible, minor frequency-resistant allele, or major frequency-resistant allele. It then predicts a resistant phenotype for samples containing resistant alleles of sufficiently high frequency, where ‘sufficiently high' is estimated using the training set. Note that for antibiotics where resistance is mediated by genes on variable copy-number plasmids (erythromycin and tetracycline) a minor population with high copy number of a resistance-carrying plasmid may sometimes be called as major resistant. After using the training data set to estimate parameters for our statistical model (see Methods section, and for results on training set, see Supplementary Table 2), we applied Mykrobe predictor to the validation set.

Figure 2c,d shows in red the false-negative calls (panel **c**) and false-positive calls (panel d) for Mykrobe predictor and the two laboratory methods: disc and Phoenix. If we consider Fig. 2c first, and focus on the 7 drugs with more than 10 resistant samples, then Mykrobe predictor misses fewer resistant calls than the other individual phenotypic methods for all drugs except ciprofloxacin. Ciprofloxacin had a false-negative rate of 4.6%; we were unable to determine the reason for these missed resistant predictions, although we note that disc and Phoenix had similar problems, and that this drug has at least one uncharacterized mechanism for resistance^16^. For the three drugs (methicillin, penicillin and erythromycin) for which our study has enough resistant samples to meet US Food and Drug Administration (FDA) criteria (false-negative rate <1.5%, upper 95% confidence interval <7.5%), Mykrobe predictor met these criteria^17^. The data underlying this plot are presented in Table 1. The results for the disc and Phoenix tests on the validation set are in Supplementary Tables 3 and 4.

The equivalent plot for false-positive calls is shown in Fig. 2d. For all drugs except penicillin and methicillin, all methods have low false-positive rates, below the FDA threshold of 3%. All methods had unacceptably high error rates for penicillin (11.7% for Mykrobe predictor, 15.1% for disc and 16.0% for Phoenix). However, it is known that for penicillin, phenotyping methods may under-detect resistance^18^,^19^,^20^, and so the apparent high false-positive rate is likely to be artefactual—that is, under-detecting resistance by disc, Phoenix and nitrocefin might lead to an (incorrect) consensus susceptible call. Indeed it has been previously shown^3^ that for some samples with weak beta-lactamase activity (exhibited by a very slowly developing nitrocefin test), resistance was not detected by either disc or Phoenix. Mykrobe predictor had an acceptable false-positive rate for methicillin of 0.0%, compared with disc (0.5%) and Phoenix (18.9%). This high false-positive rate for Phoenix was unexpected; either both disc and Etest under-detected resistance (they are both diffusion methods and might have correlated errors), or these were indeed false calls from Phoenix.

For comparison with a commercial software package, we also ran SeqSphere^6^ on the validation set, which made predictions for six drugs where resistance was gene based. Since SeqSphere predicted all 471 samples to be resistant to erythromycin and clindamycin, we excluded these drugs. Other results were broadly comparable to Mykrobe predictor and the phenotyping methods. See Supplementary Table 5 and Supplementary Figs 4 and 5 for full results.

**Figure 3:**
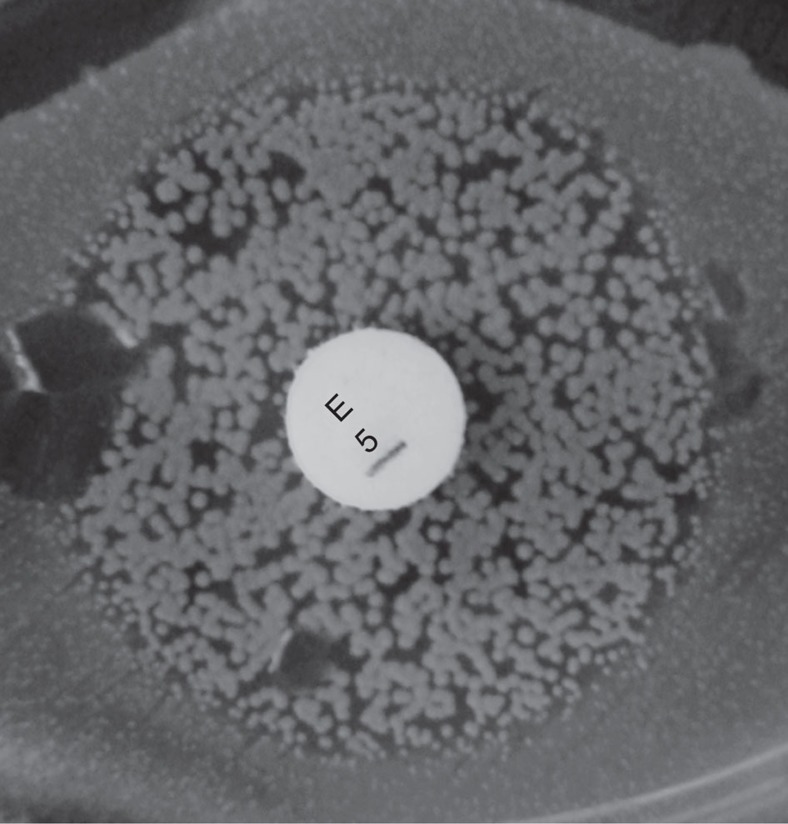
Photograph of BSAC disc test showing heteroresistant phenotype. Seen on re-running Erythromycin disc test on a sample (accession: ERS398183) where Mykrobe predictor had called a false positive (resistant) that neither disc nor Phoenix had called.

Finally, our strong prior expectation was that there would be limited within-sample diversity, due to blood culture followed by storage processes, and removal of contaminated samples (see Methods section). Mykrobe predictor confirmed this expectation and made only 6 minor calls out of 11,592 (12 drugs times 966 training and validation samples). However, we noted with interest that for the four samples where Mykrobe predictor made false-positive (major) resistant calls that were not made by disc or Phoenix, re-running the disc test resulted in contradictory results. Two changed to resistant (ciprofloxacin and erythromycin) and two produced heteroresistant phenotypes (erythromycin, tetracycline, see Fig. 3). This behaviour is consistent with a disc test presented with mixed strains or with variable plasmid loss (that would explain the three erythromycin/tetracycline results). All four samples had low levels of chromosomal diversity (between 12 and 25 ‘heterozygous' SNPs, Methods section), ruling out contamination unless by a closely related strain.

### Simulating minor infections with empirical data

**Figure 4:**
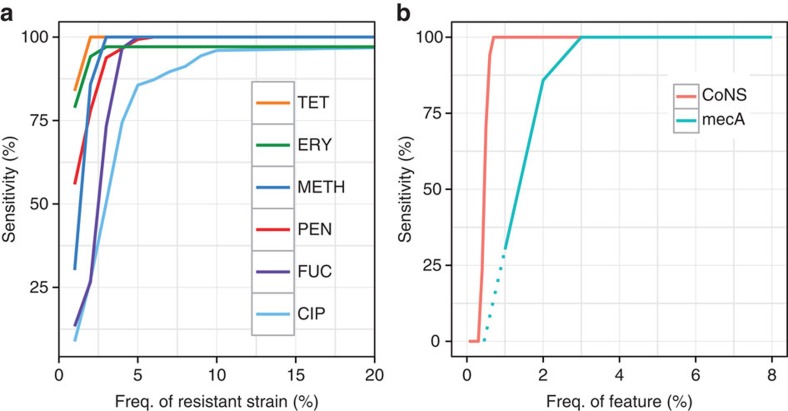
Power to detect minor populations. (**a**) Simulation 2: power to detect minor resistant alleles in 27,000 *in silico* mixtures created by taking 1,000 pairs of *S*. *aureus* samples and mixing each pair in 27 different ratios. As above, we do not estimate false-negative rates for drugs where we have <10 resistant samples, as confidence intervals would be unreasonably large. Power is greatest for the drugs where resistance genes reside on multi-copy plasmids, namely erythromycin and tetracycline. Tet, tetracycline; Ery, erythromycin; Meth, methicillin; Pen, penicillin; Fuc, fusidic acid; Cip, ciprofloxacin. (**b**) Power to detect low-frequency coagulase-negative species (red, simulation 1, *N*=540, described above) is consistently higher than power to detect *mecA* (blue, simulation 2, *N*=27,000, frequencies down to 1% only due to large sample numbers; dotted lines extrapolate linearly from points at 1 and 2%), which causes methicillin resistance in *S*. *aureus*. Thus, the risk of detecting *mecA* but not detecting the coagulase-negative species it comes from is limited.

To determine the power of our method to detect minor resistant populations, we ran Mykrobe predictor on 27,000 *in silico* ‘mixed infections', created by mixing sequence data from different samples (simulation 2, see Methods section). We show in Fig. 4a the power to detect alleles at low frequency. Our method has greatest power to detect resistance genes that lie on multi-copy plasmids, with detection power reaching 94 and 100% by the time the population frequency is 2% for tetracycline and erythromycin, respectively. For other drugs, detection power exceeds 90% once the subpopulation exceeds 8% frequency. This ability to genotype low-frequency alleles did not come at the cost of false-positive phenotypic resistance predictions—apart from the few false-positive calls that Mykrobe predictor made in pure samples (Table 1), there were no additional false positives out of 189,000 calls (27,000 mixtures × 7 drugs).

One goal for Mykrobe predictor was to avoid the combination of detecting *mecA* from *mecA*-containing-CoNS while failing to detect the CoNS species itself—thus causing a miscall of MRSA. We therefore used the simulated mixtures of *S*. *aureus* and CoNS (simulation 1), to estimate the discovery power for low-frequency CoNS species, and compared with that for low-frequency *mecA* in simulation 2—results are shown in Fig. 4b. We were able to confirm that in these mixtures such miscalls were indeed unlikely. At 1% frequency, the estimated power to detect the presence of a CoNS species was 100% (red curve), but power to detect *mecA* was 0.33 (blue curve). Above 3% frequency, power to detect each was 100%.

### Virulence elements

Antimicrobial resistance is not the only medically relevant phenotype that might be revealed by sequencing. *S*. *aureus* has a large number of virulence elements that might prove valuable to genotype. As an example, we considered Panton–Valentine leukocidin, a cytotoxin that kills leukocytes and is associated with tissue necrosis^21^. We incorporated tests for presence of the Panton–Valentine Leukocidin genes *lukPV-S* and *lukPV-K* into Mykrobe predictor and applied to sequence data from 67 *S*. *aureus* clinical isolates from an outbreak (Supplementary Data 5). The results were 100% concordant with PCR tests for the presence of these genes (23 negative and 44 positive).

### Identification of mycobacterial species in clinical samples

Species within the *M*. *tuberculosis* complex (MTBC) cause tuberculosis, but clinical samples may be other mycobacterial species. We defined probes (described in Methods section) for detection of four MTBC species (*M*. *tuberculosis*, *M*. *bovis*, *M*. *africanum* and *M*. *caprae*) and 40 nontuberculous mycobacteria (NTM) species including *M*. *abscessus*, *M*. *avium* and *M*. *intracellulare*. Co-infection with both MTBC and NTM, which is known to occur^22^,^23^, would be reported if present. We also use SNPs that have been defined in a previous publication^24^ to identify lineages within the MTBC. In terms of desired error profile, the main aim would be to minimize misclassifying a MTBC as a NTM, or vice versa. Misidentifying species within MTBC has limited impact on choice of treatment, except that *M*. *bovis* is known to be intrinsically resistant to pyrazinamide and some substrains of the Bacille Calmette–Guérin strain of *M*. *bovis* are known to be resistant to isoniazid.

**Figure 5:**
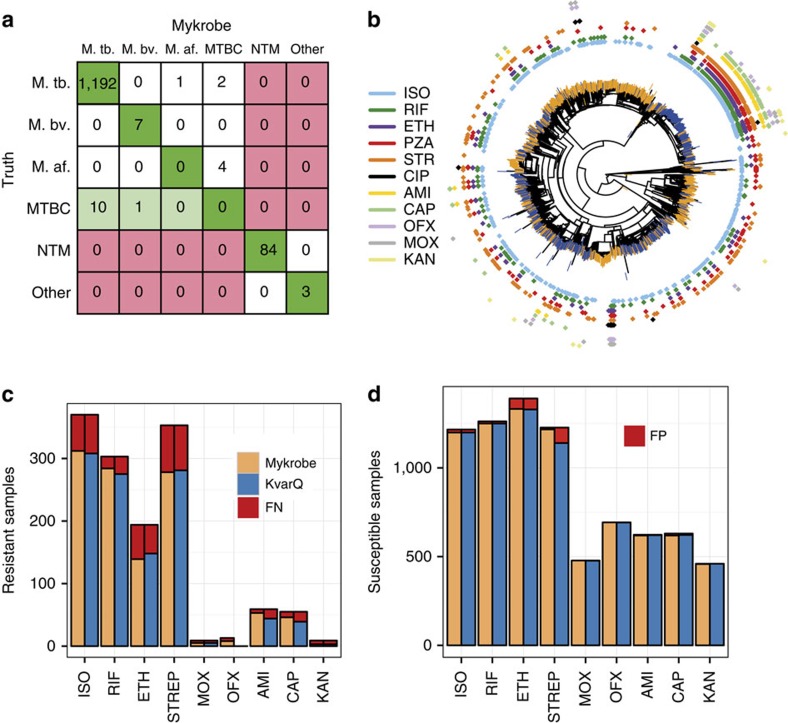
Species predictions for mycobacteria and resistance predictions for MTBC. (**a**) Species classification results on a validation set (MTBC_A2+Myco_Retro, *n*=1,304). Colours indicate misclassifications between NTM/MTBC (red), concordance with ‘truth' (dark green), or greater resolution from Mykrobe predictor than PCR (light green). M.tb., *M*. *tuberculosis*; M.af., *M*. *africum*; M.bv., *M*. *bovis*. See Supplementary Table 1 for details of ‘truth' species. (**b**) Phylogeny of MTBC samples with phenotype data, with tips marked orange or blue to indicate training set (MTBC_A, *n*=1,920) or validation set (MTBC_B, *n*=1,609). Drug resistance is shown in concentric rings around the phylogenetic tree. Resistance exists across the phylogeny, especially against isoniazid (light blue), with a clustering of multi-drug resistance in the Beijing lineage. (**c**) Proportion of resistant MTBC samples correctly identified as resistant by Mykrobe predictor (yellow) and KvarQ (light blue) compared with DST phenotype—false negatives in red. (**d**) As **c**, but showing proportion of susceptible samples called as susceptible—false positives in red. ISO, isoniazid; RIF, rifampicin; ETH, ethambutol; STREP, streptomycin; MOX, moxifloxacin; OFX, ofloxacin; AMI, amikacin; CAP, capreomycin; KAN, kanamycin.

We then evaluated species prediction on the union of data sets MTBC_A2 (1157 MTBC clinical isolates, Supplementary Data 7) and Myco_Retro (147 Mycobacterial isolates, Supplementary Data 10), where species had been identified by Hain assay, showing results in Fig. 5a. No samples were misclassified between MTBC and NTM, but there were four *M*. *africanum* and two *M*. *tuberculosis* samples that were only resolved to MTBC, and one *M*. *tuberculosis* sample misidentified as *M*. *africanum*. See Supplementary Table 6 for full results. Finally we tested our identification of the lineages as defined by Gagneux and co-workers in ref. 25 by comparing with the lineage as identified by their own tool, KvarQ^11^ on dataset MTBC_A2 and found 100% concordance.

### *M. tuberculosis* predictions match commercial assays

We use a ‘training' data set MTBC_A of 1,920 MTBC isolates with Illumina sequence data and associated drug-susceptibility test (DST) data (see Methods section, Supplementary Fig. 1 and Supplementary Table 1 with accession codes in Supplementary Data 6–8) from Oxfordshire, Birmingham, Sierra Leone and South Africa to fit the frequency threshold of 10%, above which a resistance allele is modelled as causing phenotypic resistance (see Methods section). We used an equivalent separate data set (MTBC_B, Supplementary Data 9) of 1,609 further isolates from Uzbekistan, Germany, South Africa and the UK to validate. All these samples had been previously collected for an independent study^26^ on the discovery of mutations predictive of resistance.

Figure 5b shows a phylogeny of these samples, with training and validation samples coloured at the branch tips in orange and blue, respectively. The validation set does show some clustering within the phylogeny, due to the large number of samples from Uzbekistan in the validation set, with a resulting higher number of XDR TB samples in the validation set.

Our understanding of the genetic basis for resistance in MTBC is incomplete. Common resistance mutations are on commercial line-probe assays, and explain ∼85–95% of observed resistance to the two primary first-line drugs (isoniazid and rifampicin)^27^,^28^,^29^. These assays have lower sensitivity for the third first-line drug (ethambutol) and second-line drugs^30^, and do not attempt to predict resistance for the fourth first-line drug (pyrazinamide), which is poorly understood. We built a panel of resistance mutations based on the Hain and AID line-probe assays, with a small number of additional mutations from the literature (see Methods section for details). For comparison with a method using a similar panel but without minor calls, we also ran the KvarQ tool^11^.

Figure 5c,d shows the proportion of resistant and susceptible samples that were called correctly for each drug. As expected, for first-line drugs rifampicin, isoniazid and ethambutol, the two methods (Mykrobe predictor and KvarQ) have similar power to detect resistance (93.7%, 84.3%, 71.6% versus 90.8%, 83.2%, 76.3%) and similar false-positive rates (1.0%, 1.4%, 4.2% versus 1.0%, 1.4%, 4.5%)—in line with expected performance of the Hain assay (Supplementary Figs 6 and 7).

Fewer samples were phenotyped for second-line drugs, but Mykrobe predictor had noticeably higher sensitivity for amikacin and capreomycin (89.8% and 83.6%, respectively) than KvarQ (74.6 and 70.9%), see below. There were very few false calls for second-line drugs for either method, except for a high (7%) error rate for KvarQ for streptomycin. See Supplementary Tables 7–10 for full results.

### Slow-growth *rpoB* SNPs and limitations of the gold standard

There were 12 false-positive rifampicin-resistance calls, all major calls in the gene *rpoB*. Three were L452P mutations, known to cause only low-level resistance^31^,^32^. However, on examination, we found the remaining nine calls may reflect limitations of gold-standard culture-based phenotyping. These were either mutations known to slow growth (S450L (*n*=3)^33^, S450W (*n*=1)^33^,^34^, Q432 (*n*=1)^33^,^35^), or overlap a 10 bp deletion affecting growth (Q429H (*n*=1), L430P (*n*=3)^36^). Since the proportion method used in *M*. *tuberculosis* susceptibility tests fundamentally measures growth rate as a proxy for resistance^37^, slow growth can lead to false susceptible DST results for samples with these mutations^38^,^39^,^40^,^41^. Thus, 75% of the false-positive rifampicin calls from Mykrobe predictor may actually be resistant *in vivo*, but called susceptible by DST due to the nature of the test. Indeed there is evidence that such *rpoB* mutations may be associated with poor outcome^42^,^43^.

### Minor alleles increase power to distinguish XDR from MDR TB

Mykrobe predictor predicted 56 samples were phenotypically resistant owing to minor alleles, across the 9 drugs and 1,609 samples in the MTBC_B validation set (Supplementary Data 9). Whole-genome analysis of these samples (see Methods section) found a median of 16 heterozygous sites per sample, consistent with mixed infections (local transmission or in-host evolution)^44^, although we cannot exclude the possibility of contamination with a closely related strain.

**Figure 6:**
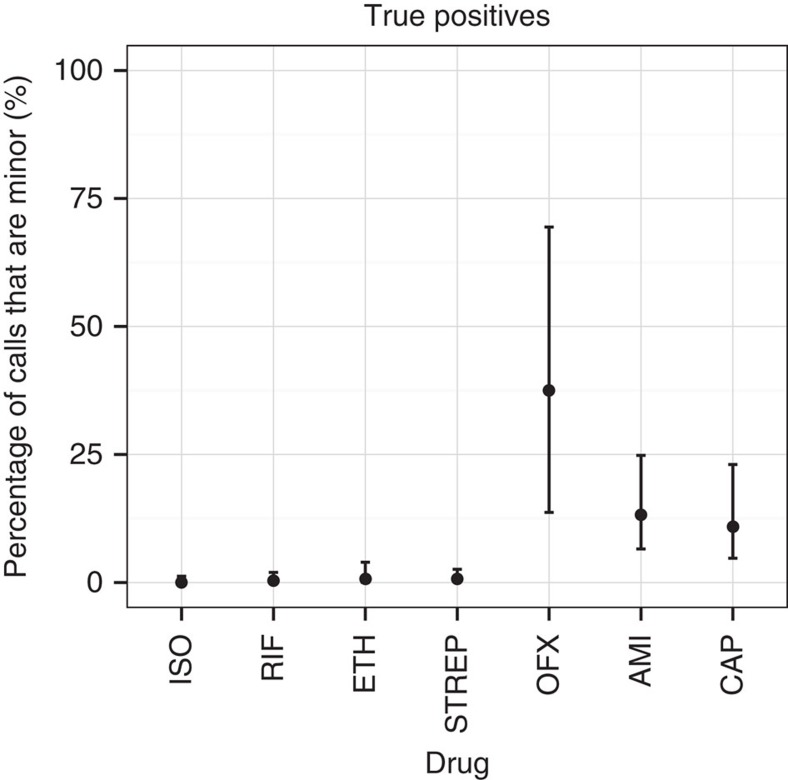
Percentage of true positive resistant calls in *M. tuberculosis* validation set due to minor alleles. Confidence intervals are calculated using the Clopper–Pearson interval. Drugs with <10 resistant samples were excluded to avoid overly large confidence intervals. For aminoglycosides and quinolones, minor populations explain between 11–38% of true positive resistance predictions. ISO, isoniazid; RIF, rifampicin; ETH, ethambutol; STREP, streptomycin; OFX, ofloxacin; AMI, amikacin; CAP, capreomycin.

We show in Fig. 6 the proportion of true positives due to minor resistant calls in the validation set, showing a clear demarcation between first- and second-line drugs. Power to predict phenotypic resistance to second-line drugs amikacin and capreomycin was significantly increased by the detection of minor populations: from 74.5 to 83.6% for capreomycin and 78.0 to 89.8% for amikacin. In addition, although here the numbers were small (*N*=13), power increased from 38.5 to 61.5% for ofloxacin. This increase in sensitivity did not come at the price of a loss of specificity. However, the effect was only seen in our validation set that had the majority of XDR samples in our data—these minor alleles were mostly (27/39) found in validation samples from Uzbekistan.

### Nanopore sequencing of *S*. *aureus*

Having evaluated performance thoroughly on two species using Illumina data, we tested Mykrobe predictor on data from the ONT MinION single-molecule sequencing machine. Since the per-base error rate is high (between 10 and 30% per base, depending on whether the molecule has been sequenced in one or two directions, termed ‘1d reads' and ‘2d reads', respectively), we modified Mykrobe predictor to expect an error rate of 10%, and to ignore the quality score for ONT data. We took a MDR *S*. *aureus* isolate from a clinical sample taken in 2014, and sequenced its genome with both the Illumina MiSeq and a ONT MinION (see Methods section for details) and ran Mykrobe predictor. The MiSeq run took 24 h and produced after cutting reads at bases with quality below 10, 368x of 122 bp reads. The MinION run took 24 h and generated 39x of ‘2d' reads, with min/mean/max length 113 bp/4.7 kb/48 kb. In both cases, Mykrobe predictor correctly predicted that the sample was resistant to penicillin, methicillin, gentamicin, trimethoprim, erythromycin, ciprofloxacin and clindamycin, and susceptible to fusidic acid, rifampicin, tetracycline, vancomycin and mupirocin. All of the resistance calls were due to detection of genes, except for ciprofloxacin where a S->L mutation at position 84 in the gene *gyr* was detected. No false-positive resistance SNPs were called. Furthermore, by truncating the MinION output file we showed that these results could have been obtained with just 7 h of sequencing.

### Software performance and usability

Mykrobe predictor memory use is comparable with that typically used by a web browser such as Chrome with multiple tabs open, and CPU requirements are low. Mykrobe predictor has been run on a Google Nexus 10 tablet, a Samsung Core Duos phone and a Raspberry Pi Model B. We give in Table 2 some performance statistics for *S*. *aureus* (abbreviated as Sa) and *M*. *tuberculosis* (abbreviated as Mtb), with comparison data from alternative tools SeqSphere^6^ (which uses whole-genome assembly), and KvarQ^11^ (which uses kmer detection).

## Discussion

Rapid determination of antimicrobial resistance profile is of critical importance to patient care for many serious bacterial infections and has wider implications for determination of treatment protocols and national surveillance. We have developed a software application, extensible to many bacterial species, called Mykrobe predictor, which can identify species, resistance profile and other genomic features such as virulence elements and phylogenetic lineage, within 3 min on a standard laptop. We have provided two implementations, for *S*. *aureus* and *M*. *tuberculosis*, and validated them extensively against clinical gold standards. Our results for *S*. *aureus* (overall sensitivity and specificity above 99%) are comparable to or better than phenotyping methods (BSAC disc test, Phoenix). For *M*. *tuberculosis*, specificity is high (98.5%) and sensitivity of 82.6% matches the line-probe assays from which our resistance panel was constructed, but still is below that of the gold standard of DST based on solid (Löwenstein–Jensen) culture. However, as new resistance-causing mutations are determined, Mykrobe predictor can be easily updated and tested, and unlike a line-probe assay or the automated Xpert-Mtb/Rif (Cepheid) assay, is unaffected by number of resistance-causing mutations in the panel.

**Figure 7:**
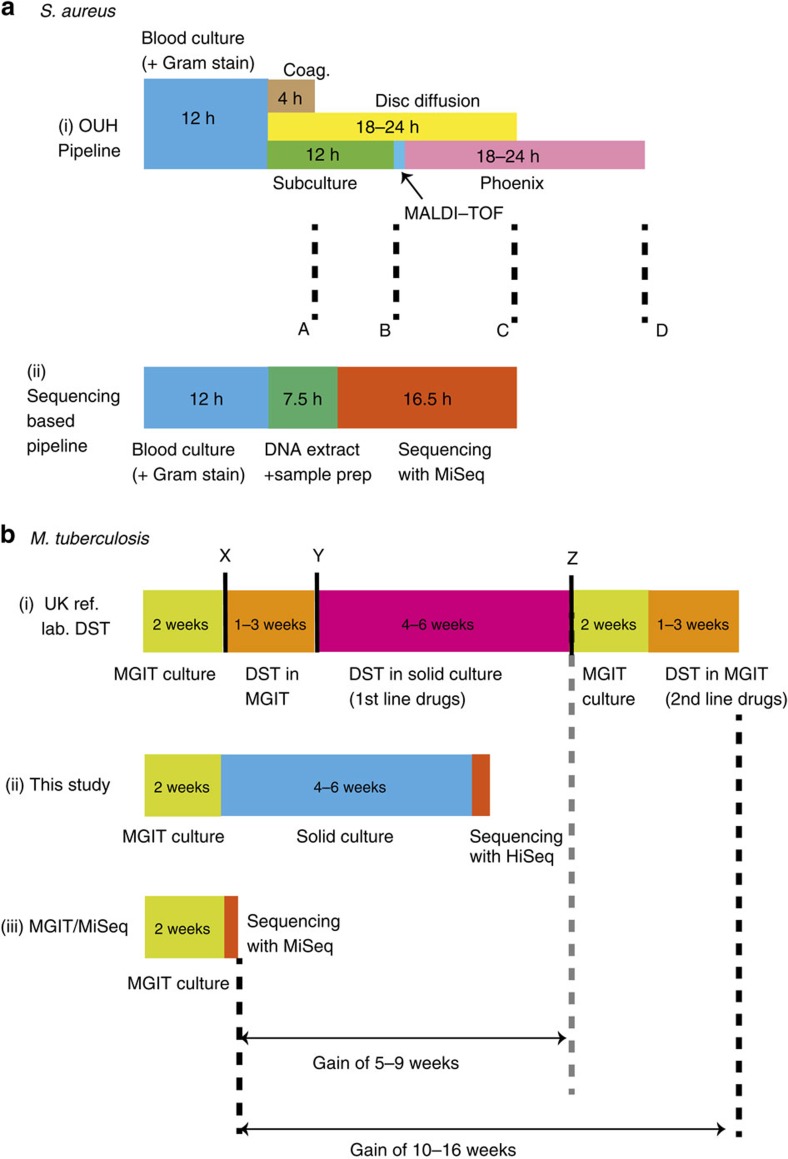
Timelines for sequencing-based analysis and culture-based DST. The timelines are shown for (**a**) *S*. *aureus* and (**b**) *M*. *tuberculosis*. In (**a**) both culture-based (**a**,i) and sequencing-based (**a**,ii) options involve 12 h of blood culture. After this, the culture-based approach (at Oxford University Hospitals clinical laboratory) follows with a direct coagulase test (Coag.) that provides a presumptive species identification at 4 h (marked ‘A'). Concurrently, blood culture is subcultured to blood agar, and MALDI-TOF confirms the species at 12 h (‘B'). A disc diffusion test for five antimicrobials (including methicillin) is performed directly from a positive blood culture providing first-line susceptibility information 18–24 h later (‘C'), assuming an acceptable inoculum. Finally, post-subculture samples are undergo extended susceptibility testing by automated broth microdilution (brandname ‘Phoenix'), giving final results after another 18–24 h (‘D'). For the sequencing-based workflow (**a**,ii), the DNA extraction plus sample preparation takes 7.5 h because samples are from blood culture, not colony isolates. With the Illumina MiSeq v3 reagents, a 16.5 h run is possible (giving paired 75 bp reads, adequate for this purpose), giving full susceptibility results at the same time as direct disc tests provide results for five drugs. (**b**) The culture-based process (**b**,i; in a typical UK reference laboratory) starts with two weeks of mycobacterial growth indicator tube (MGIT) culture, followed by a species identification test (‘X'). If the species belongs to the MTBC, then DST is run in MGIT, and at decision point ‘Y', if the sample tests susceptible to all first-line drugs, no further testing is done. MGIT DST is repeated for pyrazinamide if the first test revealed resistance to this drug. If there is resistance to any other drug, then solid culture DST is performed. If these tests show there is resistance to rifampicin then another round of MGIT culture followed by MGIT DST is done for second-line drugs. For sequencing-based approaches we show timelines for the present study (**b**,ii) and a potential alternative (**b**,iii), which would reduce time-to-results to just over 2 weeks.

We detail in Fig. 7a how the timelines for a proposed *S. aureus* sequencing workflow would compare with the clinical protocols implemented at Oxford University Hospitals clinical laboratory. Using an Illumina MiSeq 16.5 h run, our proposed sequencing workflow would provide a full set of predictions for all drugs at 36 h, ahead of Oxford University Hospital by 12 h. At other institutions, one might use MALDI-TOF or alternate rapid methods to identify the species or even methicillin resistance directly on blood culture^45^,^46^ but these cannot give the full susceptibility profile (nor any information on ancestry, epidemiology or virulence).

The acceleration provided by sequencing is even greater for *M*. *tuberculosis*, where the standard process to run first-line drug tests and, if necessary, the second-line tests afterwards, takes months (Fig. 7b). In principle these could be run in parallel, but the cost is prohibitive. By contrast, for the sequencing workflow, most clinical isolates become MGIT positive within 2 weeks. Thus, if sample preparation and sequencing is completed within 2 days of positivity^47^, one can get results in 2 weeks. This is a gain of somewhere between 5 and 17 weeks compared with gold-standard DST, depending on whether the sample is resistant to first-line drugs.

We have demonstrated that detection of simple minor resistant infections can be achieved in a robust and automated fashion, assuming at most two strains are present. We are unaware of any other resistance prediction tool that allows this. In our study, Mykrobe predictor classified 3.5%/4.9% of our *M*. *tuberculosis* training/validation samples as having minor resistant populations, with median frequency of 6.8%/9.2%, respectively. However, to match the results of the *in vitro* gold standard, we only predicted a resistant phenotype for alleles above 10% frequency. It remains an open question as to whether the lower frequency alleles have a bearing on patient outcome, despite generally failing to cause *in vitro* resistance.

For TB, initial treatment is with the preferred set of four first-line drugs (rifampicin, isoniazid, ethambutol and pyrazinamide) until it is identified that a drug-resistant strain is present, when treatment is changed to second-line drugs, many of which are less effective, less well-tolerated and more difficult to administer. The spread of MDR TB^48^ (resistant to rifampicin and isoniazid) and of XDR TB (MDR plus resistant to specific second-line drugs, that is, quinolones and an ‘injectable' such as capreomycin or amikacin) is a major global health concern. In our validation set, which contained the bulk of our XDR samples, we found minor alleles improved power to predict resistance by 9.1/11.8/23.0% for second-line drugs capreomycin, amikacin, and ofloxacin respectively (also see Fig. 6). Thus, minor alleles (or heteroresistance) may have a significant role in distinguishing MDR from XDR TB. However, since only 18%/44% of our training/validation samples were phenotyped for at least one quinolone or injectable, this finding needs replication with larger data sets.

Recognizing that lack of bioinformatics expertise is a barrier to clinical adoption, we provide drag-and-drop Windows and Mac applications (see screenshots in Supplementary Figs 8–10) and a linux version that could, for example, enable a cloud service. Our demonstration that Mykrobe predictor can work on low-specification hardware, such as a mobile phone or Raspberry Pi is intended to enable future applications of resistance determination in the field, in low-resource settings with no internet access. Along these lines, the advent of portable single-molecule sequencing machines that deliver long-read information in real time will change the face of clinical microbiology. The ability to sequence a single sample removes the need to batch samples until an Illumina MiSeq sequencing run is justified, reducing bedside-to-treatment time, and the long reads could provide vital information on mixed infection composition. Our *N*=1 test of the Oxford Nanopore MinION machine offers only proof-of-principle, but unlike one recent report^49^, we were able to get fully concordant results for both gene and SNP-driven resistance with only 13 × coverage, without the high per-base error rate causing any false SNP calls. Since Mykrobe predictor can test the de Bruijn graph and assess confidence of resistance/susceptibility approximately an order of magnitude faster than MinION reads arrive, this could be done as the reads come in from the machine, enabling a real-time decision to be made as to whether to stop sequencing. In this case we could have stopped after 7 h of sequencing, and throughput on this maturing platform has improved considerably since then.

In terms of the path to clinical use of Mykrobe predictor, the next steps for *S*. *aureus* and *M*. *tuberculosis* are further clinical testing, and then obtaining regulatory approval. We will be running Mykrobe predictor in parallel with the clinical workflow in hospitals in Oxford, Leeds and Brighton (UK) for 3 months starting in 2015 as part of a Health Innovation Challenge Fund project. It is relatively simple to extend Mykrobe predictor to other bacterial species by using a panel of known sites and genes, as we did in this study. However, more generally, for a species where resistance mechanisms are poorly characterized, one would need to use a large training set with both whole-genome sequence data and phenotype information for hypothesis-free discovery of causal mutations or purely predictive markers. Some such studies have been done^50^,^51^, and we expect many more.

There are two main limitations to the current implementation of Mykrobe predictor. First, we suspect that incorporating a more general model of mixtures, rather than simply major/minor clones, will be of value when analysing *M*. *tuberculosis* samples direct from sputum. Second, our sensitivity for *M*. *tuberculosis* is low (82.6% across all drugs) compared with traditional DST, and completely excludes the first-line drug pyrazinamide since known mutations are poorly predictive. This issue, shared by all molecular assays, can only be resolved by large-scale sequencing and phenotyping studies.

## Methods

### Study design

The objectives of this study were:

- To show that our software program could deliver automated antimicrobial-resistance predictions for two bacterial species given a pre-specified genotype-to-drug-resistance mapping. The limitations of the pre-specified mapping would place an upper bound on sensitivity—for *S*. *aureus* that upper bound was above 99%, but for *M*. *tuberculosis* we followed the HAIN and AID assays, expecting sensitivity of ∼82%. To achieve this, we used independent training and validation sets previously obtained in other studies^3^,^26^. For both species, the number of samples with resistant phenotypes was limiting, and we only estimated false-negative rates where there were sufficiently many (>10) resistant samples in the validation set, reporting confidence intervals calculated using the Clopper–Pearson interval.
- To handle contamination and mixture issues seen in clinical samples. We used independent data sets for design of probes and validation. To include some realistic sampling of species, the validation set for mycobacteria included the set Myc_Retro consisting of all mycobacterial (meaning positive MGIT culture) samples sent to the laboratories at the Oxford John Radcliffe Hospital between 2 June 2013 and 29 January 2014.
- To observe whether minor resistant population detection could increase predictive power of phenotypic resistance without compromising specificity in the data sets collected.

All data sets used are described in Supplementary Fig. 2 and Supplementary Table 1 with full sample information in Supplementary Data 1–11.

### Phenotyping and sequencing of *S*. *aureus* data sets

Initial phenotyping of the training and validation sets was described in detail in Gordon *et al*.^3^ The training set was phenotyped using either the Vitek automated system (bioMerieux) or the Stokes method disc diffusion^52^, whereas all validation samples were phenotyped using two methods: a BSAC disc test^15^ and the Phoenix automated microbiology system (BD Biosciences). For trimethoprim only disc testing was performed.

We removed 6 samples from the training data set and 20 samples from the validation set that were contaminated (see section Contamination in *S. aureus* genome sequence data below for details). All samples where there was discordance between Phoenix and disc for any drug in Gordon *et al*.^3^ were rerun on Phoenix (for all drugs) and previous results from Gordon *et al*.^3^ were discarded.

Samples were sequenced on Illumina HiSeq 2000 platform, with mean read length (after cutting reads at bases with quality score below 10) of 87 bp and mean depth of 87, as described in ref. 3.

### Species identification in general

To ensure sensitivity to low-frequency contaminating species, we wanted to use more than one probe (as opposed to common methods based on single genes, for example, *rpoB*, *gyrA* and so on.), which contained more sequence than a single gene. We developed a system for designing a hierarchy of markers (contigs) that first separated two phylogroups (*S*. *aureus* from CoNS, or MTBC from NTM), and then identified species present within a phylo group. We first built a Bruijn graph by pooling several hundred samples from both phylogroups, and pulled out all unique and unambiguous contigs (‘unitigs') and calculated the frequency of each contig in each phylo group. We chose the most highly differentiated contigs to form marker panels to distinguish the groups. This process was then run again to find contigs informative at the species level.

### Identification of staphylococcal species

The above process was applied to 731 staphylococcal isolates in training set St_A, which combines data sets St_A1 (532 clinical *S*. *aureus* isolates, Supplementary Data 1) and St_A2 (199 CoNS isolates, Supplementary Data 2) , using Cortex^13^ with kmer size 15, to produce probes (contigs) for phylogroups (*S*. *aureus* versus coagulase-negative staphylococci) and species (*S*. *aureus*, *S*. *epidermidis*, *S*. *haemolyticus*, other coagulase-negative species). We assume a positive Gram stain has been obtained, and use 33 alleles of the catalase gene (Supplementary Table 11) to confirm presence of staphylococci. The percentage of sequence in the probe panel found in each training sample (‘recovery') was plotted, and extreme outliers were ignored as possible errors in the SRA metadata; detection thresholds were chosen based on recovery in the training set: 90% for *S*. *aureus*, 30% for *S*. *epidermidis* and *S*. *haemolyticus*, 10% for other staphylococci and 20% for the catalase gene.

### *S*. *aureus* resistance panel

Starting from the variant catalogue described by Gordon *et al*.^3^, we made the following alterations. B434N in *fusA* was changed to D434N. Q456K in *rpoB* was removed as Q was not the amino acid at position 456 of the referenced *rpoB* gene. We added *rpoB* N474K, described by Villar *et al*.^53^ We also considered the 10 novel mutations reported by Dordel *et al*.^54^ We found three of the mutations (PBP1 H499Y, PBP2 T31M and PBP2 D156Y) in our derivation set. These were found in samples phenotypically susceptible to methicillin, so none of the variants from that paper^54^ were included in the final catalogue. Variants that changed predicted MIC (Minimum Inhibitory concentration) but did not confer resistance on their own were not included. For resistance genes we took all versions/alleles of the gene from National Center for Biotechnology Information that were not explicitly annotated as existing in a susceptible strain, and did not have stop codons. The full list of chromosomal mutations, genes and accession codes can be found in Supplementary Tables 12–14.

### Data structures for genotyping

We implemented two versions of Mykrobe predictor. The first builds a whole-genome de Bruijn graph of the sample, and then takes the intersection of this with the de Bruijn graph of all (alleles of) genes and mutations on different genetic backgrounds (the ‘target graph'). This requires ∼300 Mb of RAM for a typical Illumina data set, but could in principle grow for very large data sets. To control memory use, the second approach builds the target graph first, and then only loads sample data that intersects it, reducing RAM use to 100 Mb. Both methods give absolutely identical results, and we ran all analyses for this paper using the second approach.

### Genotyping at mutations

We use three competing models: pure susceptible, minor resistant (frequency=10*%*) and major resistant (we used frequency=75%, but we expect that values from 60–100% would result in identical model choice). In this and subsequent sections, a subscript MAJ, MIN or S refers to the Major Resistant, Minor Resistant or Susceptible models. We use the following uninformative priors:


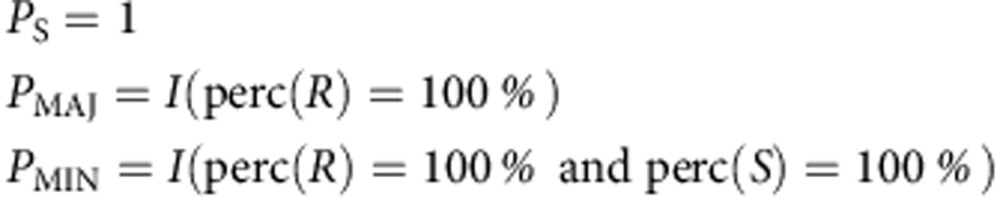


where perc(*R*) and perc(*S*) are, respectively, the percentage of the kmers in the resistant/susceptible alleles that are seen in the sample, and *I* is an indicator function. We use the following simple Poisson model for the likelihoods for all three models.

Susceptible model: likelihood specified by Poisson coverage on S allele, plus errors driving both coverage loss on *S* allele and coverage on *R* allele.


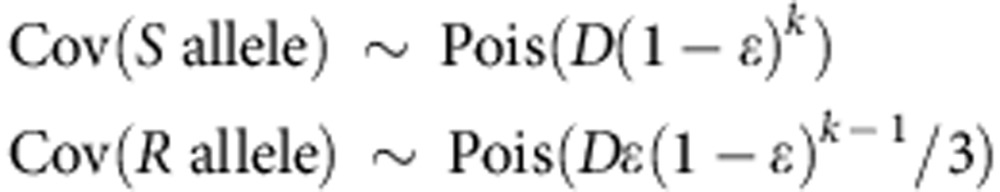


Major and minor resistant models: Poisson coverage on both alleles scaled by frequency:


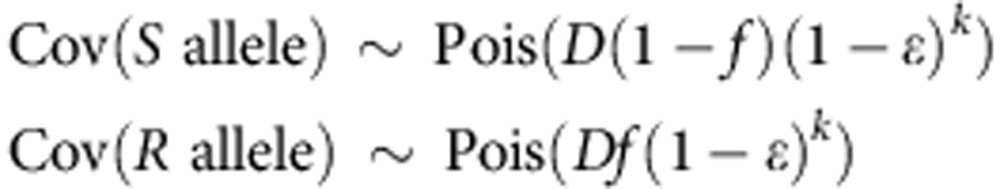


where Cov() is a function returning median coverage on an allele, *D* is the depth of coverage, *ɛ* is the per-base error rate, *κ* is the kmer size, and the frequency *f* of the resistance allele is 0.1/0.75 for the minor/major resistant model.

The vast majority of mutations in the panel result in amino acid changes, but some of the mutations occur within promoter regions. When we refer to genetic background, we simply mean mutations present in the population within one kmer length of the site of interest. For all mutations in the panel, we run through all genetic backgrounds, and if appropriate, all possible nucleotide changes that would generate the specified amino acid change, and find the highest coverage resistant allele and susceptible allele. These are then passed into the three models. The Maximum A Posteriori Model is chosen.

### Resistance calling at mutations

If the frequency of the genotyped variant is below a threshold (T, determined below) we report the mutation but predict a susceptible phenotype. Otherwise we predict a resistant phenotype, and mention whether we classified this as a minor or major population.

For *S*. *aureus*, the protocol undergone by samples in the standard clinical workflow removed almost all mixture, and so there were almost no minor alleles to either train or validate on—we set an arbitrary threshold of 10%. For *M*. *tuberculosis*, we did not have enough data to estimate per-drug thresholds, and we knew that the phenotyping data was imperfect (for example *embB* mutations at amino acid 306 lead to ethambutol MICs that are very close to the critical concentration, leading to stochastic switching of test results). We examined the two frequency distributions of resistance alleles present in phenotypically resistant/susceptible samples in the training set (Supplementary Fig. 11), and selected a single threshold of 10% for all drugs. The corresponding distributions for the validation set can be seen in Supplementary Fig. 12.

### Genotyping at genes

The expected proportion of kmers in a gene that are observed is


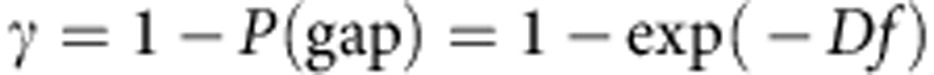


We use the following priors


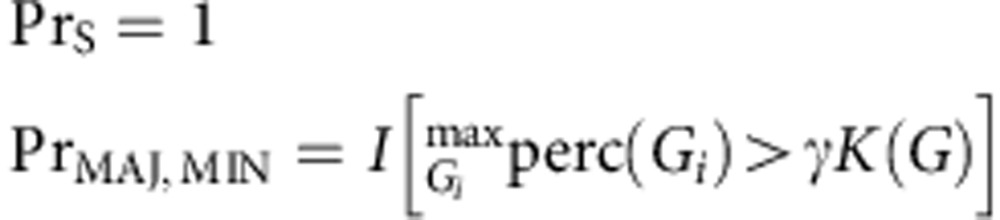


where each gene *G* has multiple exemplars *G*_*i*_ representing diversity of that gene, *I* is an indicator function, and perc() is a function returning the percentage of kmers present in the sample. *K* is the minimum percentage of kmers expected to be recovered for a gene, based on the empirical level of diversity observed in the training set.


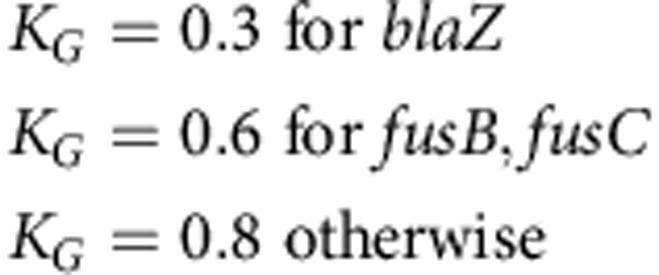


The likelihoods for major and minor models depends on the probability of having the observed median coverage across the gene. If dpois refers to the probability density of a Poisson distribution, and the observed median coverage across the gene is *m*, then the likelihood for the major and minor resistant models are given by


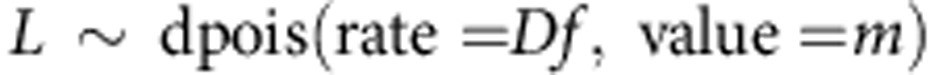


where *D* is the median depth of coverage on the species-specific probe panel defined above, *k* is kmer and *R* is read length.

### Resistance calling at genes

In the training set, a threshold frequency was chosen for each gene, such that above this frequency the sample was more likely to be resistant than sensitive. If the gene was genotyped as Minor Resistant but the gene's estimated frequency (based on median coverage over overall depth of coverage) was below this threshold a susceptible phenotype was reported. Otherwise, a (major or minor) resistant phenotype was reported.

The minimum frequency thresholds were: erythromycin: 0.19, fusidic acid: 0.03, gentamicin: 0.04, methicillin: 0.06, mupirocin: 0.21, penicillin: 0.04 , tetracycline: 0.13 and otherwise=0.03.

### Identification of mycobacterial species

We chose to identify 4 MTBC species (*M*. *tuberculosis*, *M*. *africanum*, *M*. *bovis*, *M*. *caprae*) and 40 NTM species (*M*. *abscessus*, *M*. *africanum*, *M*. *aromaticivorans*, *M*. *avium*, *M*. *bovis*, *M*. *branderi*, *M*. *caprae*, *M*. *chelonae*, *M*. *chlorophenolicum*, *M*. *chubuense*, *M*. *colombiense*, *M*. *crocinum*, *M*. *flavescens*, *M*. *fluoranthenivorans*, *M*. *fortuitum*, *M*. *gilvum*, *M*. *gordonae*, *M*. *hodleri*, *M*. *interjectum*, *M*. *intracellulare*, *M*. *kansasii*, *M*. *lentiflavum*, *M*. *leprae*, *M*. *malmoense*, *M*. *marinum*, *M*. *mucogenicum*, *M*. *pallens*, *M*. *peregrinum*, *M*. *phage*, *M*. *pyrenivorans*, *M*. *rufum*, *M*. *rutilum*, *M*. *scrofulaceum*, *M*. *senegalense*, *M*. *smegmatis*, *M*. *sphagni*, *M*. *szulgai*, *M*. *triplex*, *M*. *tuberculosis*, *M*. *tusciae*, *M*. *ulcerans*, *M*. *vaccae*, *M*. *xenopi.*). Marker panels were generated for MTBC and NTM (‘phylo groups') and for individual species within those groups, using the same method as for staphylococci based on training set consisting of data sets MTBC_A1 (338 MTBC clinical isolates, Supplementary Data 6) and Myco_SRA (380 Mycobacterial samples downloaded from the SRA, Supplementary Data 11) . The required percentage of probe sequence to be found in each of the ‘phylo group' panels was set as 70% for MTBC, 25% for the NTM panel, and 30% for all species panels. Above this threshold the phylo group or species was predicted to be present.

We also used the lineage-informative SNPs defined by Stucki *et al*.^24^ to assign *M*. *tuberculosis* lineages: Beijing/East Asia, East Africa / Indian ocean, Delhi/Central Asia, European/American, West Africa 1 and 2 or Ethiopian.

### *M*. *tuberculosis* phylogeny

We used the underlying phylogeny of samples in sets MTBC_A and MTBC_B, which was constructed using RAxML (version 8.0.5) using a GTRCAT (General Time Reversible—CAT) model^55^. For this study, we combined this tree with data set membership and phenotypic resistance metadata using the analyses of Phylogenetics And Evolution Package^55^ to produce Fig. 2b.

### *M*. *tuberculosis* resistance panel

We used a panel of MTBC resistance variants from the HAIN^29^, Cepheid^56^ and AID^57^ assays supplemented by others from the literature^58^,^59^,^60^ (Supplementary Table 15). All possible SNPs that would account for amino acid or DNA variants associated with resistance were introduced on multiple susceptible backgrounds. These backgrounds were selected as follows. Two samples were chosen from each of the six *M*. *tuberculosis* lineages. For those samples, paired-end reads were mapped by Stampy (version 1.0.17)^61^ to the H37Rv reference genome (GenBank accession code NC000962.2). SNP calls were made with SAMtools^62^ mpileup (version 0.1.18), requiring a minimum read depth of 5 and at least one read on each strand. We looked for variants in the 20 bases on either side of each resistance mutation in each of those 12 samples—these, along with the reference, defined a set of genetic backgrounds.

Since the underlying panel is almost identical, we expect Mykrobe predictor to perform equivalently to the HAIN test. Comparing on MTBC_A, Mykrobe predictor and HAIN have similar power to detect resistance (85.5%, 94.1%, 76.5% versus 85.5%, 93.5%, 76.5%) for first-line drugs rifampicin, isoniazid and ethambutol, respectively—see Supplementary Figs 5 and 6.

We chose an underlying frequency of 10% for the minor resistant model as this gave appropriately low false-positive rates when comparing with phenotypes in the training set (Supplementary Table 7).

### Software

The Mykrobe predictor software is freely available (opensource) at www.github.com/iqbal-lab/Mykrobe-predictor for non-commercial academic and research use only, under a licence from Isis Innovation, the technology transfer company of the University of Oxford. We provide a Linux command-line version and desktop ‘drag-and-drop' applications for 64-bit Windows and Mac OS X (Supplementary Software 1–4; see screenshots in Supplementary Figs 8–10 and screencasts in Supplementary Movies 1 and 2).

### Contamination in *S. aureus* genome sequence data

We removed six samples from the St_A1 and nine samples from St_B1, as BLAST of the assembly contigs confirmed presence of non-staphylococcal contamination. We also removed 11 samples from St_B1, which had >40 confident heterozygous SNPs as called by the Cortex variation assembler (independent workflow, *k*=31, ploidy=2, automatic error cleaning, using ‘bubble caller' calling algorithm). We used this threshold of 40 heterozygous sites to determine whether a sample was contaminated by an unrelated strain—less than that we considered a conceivable level of in-host diversity.

### Phylogeny of *S*. *aureus*

A conservative set of SNPs was called for each sample in the training set (St_A1) and validation set (St_B1) by first mapping reads to the MRSA252 reference genome with Stampy^61^, and then calling variants with samtools^62^. SNPs with less than five reads' support, or without at least one read on each strand, were filtered, as were multiallelic SNPs, SNPs with at least 5 reads on both alleles, and SNPs in repetitive regions. A phylogeny was then constructed using RAxML^55^ with the following command-line.

RAxML-7.7.6/raxmlHPC-PTHREADS-SSE3 -s phylipFile -n outputPrefix -m GTRCAT -p 12345 -c 1 -T 2 -D ON -f c -F ON -V.

This tree was used to produce Fig. 2b, to display the distribution of resistance training and validation samples across the phylogeny.

### Simulation 1: detecting staphylococcal contaminants

We took 18 CoNS samples from the St_B2 (Supplementary Data 4) data set (9 *S*. *epidermidis* and 9 *S*. *haemolyticus*), and 9 *S*. *aureus* samples from St_B1 (Supplementary Data 3), and associated random pairs of samples from these sets (always 1 *S*. *aureus* and 1 CoNS, mixture pairs below). We then made *in silico* mixtures of these pairs with the CoNS samples at 30 frequencies ranging from 0.5 to 20%. We ran Mykrobe predictor on all 540 mixtures to determine sensitivity to detect CoNS at each frequency. Accession identifiers of the pairs: (SRR1182410, ERR410084), (SRR1182413, ERR410093), (SRR1182415, ERR410136), (SRR1609104, ERS398139), (SRR221652, ERS398155), (SRR398319, ERS398179), (SRR496759, ERS398307), (SRR496761, ERS398353), (SRR496889, ERS398370), (ERR085178, ERR410084), (ERR085180, ERR410093), (ERR085182, ERR410136), (ERR085188, ERS398139), (ERR085190, ERS398155), (ERR085192, ERS398179), (ERR085258, ERS398307), (ERR085260, ERS398353) and (ERR085262, ERS398370).

### Simulation 2: detecting low-frequency alleles in *S. aureus*

We took 450 samples from the *S*. *aureus* data set St_B1 that all had at least 100 × mean sequencing depth of coverage across the genome, and subsampled them randomly to precisely 100 × . We then took 1,000 random pairs of samples from this set, and for each pair, combined subsets of their reads so as to create 27 different mixtures with ratios ranging from 1:99 to 99:1.

We than ran Mykrobe predictor on these mixtures, to determine the frequency at which rare SNPs/genes were detected, and to confirm that this sensitivity did not cause false-positive predictions of phenotypic resistance.

### Analysing *S. aureus* samples with discrepant disc retests

Whole-genome variant calling was done with the Cortex variation assembler^63^ (version 1.0.5.21, independent workflow, *k*=31, ploidy=2, automatic error cleaning, using ‘bubble caller' calling algorithm) with the following command: perl <cortex_dir>/scripts/calling/run_calls.pl --ref Absent --fastaq_index INDEX --first_kmer 31 --auto_cleaning yes --outdir OUTDIR --ploidy 2 --genome_size 2800000 --mem_height 20 --mem_width 100 --qthresh 10 --do_union no --logfile log.txt --workflow joint --vcftools_dir /path/to/vcftools_0.1.9/.

The number of variants which were genotyped as ‘heterozygous' with genotype confidence >1 was counted for each of these samples.

### Running other software for comparison

The commercial software SeqSphere recently demonstrated resistance gene detection^64^. The template (the SeqSphere equivalent of a gene panel) used was not publicly released but the authors were kind enough to allow us to use it. We ran SeqSphere on the sequence data from the validation set St_B1 (471 *S*. *aureus* isolates), in pipeline mode. We followed the methods in the original paper^64^ with the exception that the Velvet assembly was run by SeqSphere with the default parameters. Although used in that paper, erythromycin and, therefore, clindamycin were excluded from the comparison since all samples were called as Resistant.

KvarQ^11^ version 0.12.3a1 was run on the training and validation MTB fastq files using the provided ‘MTBC' testsuite.

### Heterozygosity in *M. tuberculosis* set MTBC_B

The standard ‘independent workflow' of Cortex was used, with the bubble caller algorithm, using the run_calls script which is part of Cortex. Parameter values: ploidy=2, do_union=yes, auto_clean=yes, kmer_size=31. Only sites with genotype confidence GT_CONF>1 were considered.

### Nanopore sequencing

The DNA library was prepared using the Genomic DNA Sequencing Kit SQK-MAP005 according the manufacturer's protocol (Version MN005_1115_revC_26Nov2014), with small modifications.

Without undergoing any shearing process, 2 μg of DNA were treated with PreCR Repair Mix (New England BioLabs, NEB) , to repair possible damage to the DNA that could interfere with the sequencing process. Following Oxford Nanopore recommendation for the optional PreCR treatment, each μg of DNA was first diluted in nuclease-free water to a volume of 85 μl to which 10 μl ThermoPol Reaction Buffer, 1 μl NAD^+^, 1 μl 10 μM dNTPs and 2 μl PreCR Repair Mix were added, and the two reactions were incubated at 37 °C for 30 min. The repaired DNA was purified with 1 volume (100 μl) Agencourt AMPure XP beads (Beckman Coulter, UK) according to manufacturer's instructions. The purified DNA from each of the two reactions was eluted from the magnetic beads in 40 μl EB buffer and pooled together. The 80 μl of DNA were end repaired using the NEBNext End Repair (NEB) module from the NEBNext DNA Library Prep Master Mix Set (New England BioLabs, UK) for Illumina by adding 10 μl buffer and 5 μl of enzyme mix, and nuclease-free water to a final volume of 100 μl, and incubating the reaction at 20 °C for 30 min. The end-repaired DNA was purified with 1 × volume (100 μl) Agencourt AMPure XP beads and the purified product eluted in 25 μl EB buffer. dA tailing on the purified DNA was performed in a final volume of 30 μl using the dA-tailing module of the NEBNext DNA Library Prep Master Mix Set for Illumina: 3 μl buffer and 2 μl Klenow fragment (3′→5′ exo^−^) were added and the reaction was incubated at 37 °C for 30 min. The dA-tailed DNA was then transferred to Eppendorf LoBind tubes. The ligation of the Oxford Nanopore adaptor was performed by adding 10 μl adaptor mix, 2 μl HP (‘hairpin' adaptor), 50 μl blunt/TA ligase master mix (NEB), and water to a final volume of 100 μl, with a 10 min incubation at room temperature. Extra care was taken to mix reagents during the ligation and the following steps only through pipetting to avoid, as much as possible, unnecessary contact of the ligated and protein-bound DNA with the tube walls.

The fragments with a hairpin ligated were selectively pulled down using Dynabeads His-Tag Isolation and Pulldown. A 10 μl aliquot of beads was washed twice using 200 μl of a 1:2 dilution of Oxford nanopore bead-binding buffer (BBB) and resuspended in 100 μl undiluted BBB before adding to the sample. After a 5 min incubation at room temperature, the tube was placed on a magnetic rack and the supernatant removed. The beads were then washed twice with 200 μl diluted BBB, and any excess of buffer was removed by aspiration with a pipette. The beads were resuspended in 25 μl Oxford Nanopore Elution Buffer and left for 10 min at room temperature. The tube was placed on a magnetic rack and the library was transferred to a new tube.

In parallel with the library preparation, the MinION was made ready for sequencing. A new flow cell (R7.3 chemistry) was loaded on the MinION and the Platform QC protocol in the MinKNOW software was run to assess the number of available pores for sequencing. At the end of the QC, the flow cell was primed by loading twice 150 μl of a mix of 75 μl Oxford nanopore-running buffer (2X), 72 μl nuclease-free water, 3 μl Fuel mix, and leaving at least a 10 min interval between subsequent loadings of buffer or library. Once the flow cell was ready, a mix of 6 μl library, 3 μl fuel mix, 75 μl Oxford nanopore-running buffer (2X), nuclease-free water to a final volume of 150 μl was loaded on the flow cell and the 48-h sequencing protocol was started. Additional aliquots of library were loaded after 4 and 24 h to increase yield.

Once the sequencing run had begun, the Metrichor program ( https://metrichor.com) was started and the raw data were automatically uploaded for base calling (workflow 2D Basecalling rev 1.14).

## Additional information

**Accession codes:** All the sequencing data is available at the European Nucleotide Archive and National Center for Biotechnology Information's SRA. Accession codes are listed, together with phenotyping data, in Supplementary Data 1–10.

**How to cite this article:** Bradley, P *et al*. Rapid antibiotic-resistance predictions from genome sequence data for *Staphylococcus aureus* and *Mycobacterium tuberculosis*. *Nat*. *Commun.* 6:10063 doi: 10.1038/ncomms10063 (2015).

## Supplementary Material

Supplementary Information Supplementary Figures 1-12 and Supplementary Tables 1-15

Supplementary Data 1 Dataset St_A1

Supplementary Data 2 Dataset St_A2

Supplementary Data 3 Dataset St_B1

Supplementary Data 4 Dataset St_B2

Supplementary Data 5 Dataset St_PVL

Supplementary Data 6 Dataset MTBC_A1

Supplementary Data 7 Dataset MTBC_A2

Supplementary Data 8 Dataset MTBC_A3

Supplementary Data 9 Dataset MTBC_B

Supplementary Data 10 Dataset Myco_retro

Supplementary Data 11 Dataset Myco_SRA

Supplementary Software 1 Windows executable for 'Mykrobe predictor' for *S. aureus*

Supplementary Software 2 Mac OS X executable for 'Mykrobe predictor' for *S. aureus*

Supplementary Software 3 Windows executable for 'Mykrobe predictor' for *M. tuberculosis*.

Supplementary Software 4 Mac OS X executable for 'Mykrobe predictor' for *M. tuberculosis*

Supplementary Movie 1 Real-time video of 'Mykrobe predictor' *S. aureus* desktop app

Supplementary Movie 2 Real-time video of 'Mykrobe predictor' *M. tuberculosis* app.

## Figures and Tables

**Table 1:** Comparison of Mykrobe predictor *S*. *aureus* resistance prediction results with consensus phenotype for validation set (St_B1).

**Drug**	**FN(R)**	**FP(S)**	**VME (95% CI)**	**ME (95% CI)**	**PPV (95% CI)**	**NPV (95% CI)**
PEN	1 (377)	11 (94)	**0.3% (0.0–1.5%)**	11.7% (6.0–20.0%)	97.2% (95.0–98.6%)	98.8% (93.5–100%)
ERY	1 (79)	0 (392)	**1.3% (0.0–6.9%)**	**0.0% (0**–**0.9%)**	100% (95.4–100%)	99.7% (98.6–100%)
CIP	3 (65)	1 (406)	4.6% (1.0–12.9%)	**0.2% (0.0–1.4%)**	98.4% (91.5–100%)	99.3% (97.9–99.8%)
METH	0 (54)	0 (417)	**0.0% (0–6.6%)**	**0.0% (0–0.9%)**	100% (93.4–100%)	100% (99.1–100%)
FUS	0 (41)	4 (430)	0.0% (0–8.6%)	**0.9% (0.3–2.4%)**	91.1% (78.8–97.5%)	100% (99.1–100%)
CLIN	0 (25)	1 (97)	0.0% (0–13.7%)	1.0% (0.0–5.6%)	96.2% (80.4–99.9%)	100% (96.2–100%)
TET	0 (17)	1 (454)	0.0% (0–19.5%)	**0.2% (0.0–1.2%)**	94.4% (72.7–99.9%)	100% (99.2–100%)
RIF	0 (5)	0 (466)	N/A	**0.0% (0–0.8%)**	N/A	100% (99.2–100%)
GEN	1 (3)	0 (468)	N/A	**0.0% (0–0.8%)**	N/A	99.8% (98.8–100%)
MUP	0 (2)	0 (348)	N/A	**0.0% (0–1.1%)**	N/A	100% (98.9–100%)
TRIM	0 (1)	1 (188)	N/A	**0.5% (0.0–2.9%)**	N/A	100% (98.0–100%)
VAN	0 (0)	0 (471)	N/A	**0.0% (0–0.8%)**	N/A	100% (99.2–100%)

CI, confidence interval; CIP, ciprofloxacin; CLIN, clindamycin; ERY, erythromycin; FDA, food and drug administration; FN, false-negative calls; FP, false positives; FUS, fusidic acid; GEN, gentamicin; ME, major error rate (false-positive rate); METH, methicillin; MUP, mupirocin; N/A, not applicable; NPV, negative predictive value; PEN, penicillin; PPV, positive predictive value; R, total number of resistant samples; RIF, rifampicin; S, total number of susceptible samples; TET, tetracycline; TRIM, trimethoprim; VAN, vancomycin; VME, very major error rate (false-negative rate).

Resistance prediction results for Mykrobe predictor on the *S*. *aureus* validation set (St_B1), treating the consensus phenotype as gold standard except for trimethoprim (which Phoenix does not test) where the disc test was used as truth. VME only shown where R>10. ME only shown where S>10. Error rates shown with 95% CI calculated by Clopper–Pearson. Error rates meeting the FDA requirements are in bold.

**Table 2:** Performance and feature comparison of Mykrobe predictor, *SeqSphere* and *KvarQ* software.

	**Mykrobe predictor (Sa)**	**SeqSphere (Sa)**	**Mykrobe predictor (Mtb)**	**KvarQ (Mtb)**
RAM	100 Mb	8 Gb	100 Mb	30 Mb
Time/sample on laptop	1.5 min	—	2.75 min	40 min
Time/sample on server	44 s	19 min	47 s	23 min
CPU time (Mtb validation set)	—	—	1 day	30 days
CPU time (Sa validation set)	5.8 h	6.2 days	—	—
Speciation of clinical samples	Yes	No	Yes	No (MTBC only)

MTBC, *Mycobacterium tuberculosis* complex; Mtb, *Mycobacterium tuberculosis*; Sa, *Staphylococcus aureus*.

We show elapsed time for one sample on a laptop (Macbook Air with 8 GB RAM) and a server (Dell PowerEdge R820 with 32 cores, 1 Tb RAM), and then for the entire *S*. *aureus* and *M*. *tuberculosis* validation sets St_B1_ (*n*=491) and MTBC_B_ (*n*=1609) respectively. We ran KvarQ on 1 thread for ease of parallelization and comparison, as recommended by the authors. However, we ran SeqSphere on 4 threads because to use one would have taken a prohibitively long time.
