# Corrigendum: Rapid antibiotic-resistance predictions from genome sequence data for *Staphylococcus aureus* and *Mycobacterium tuberculosis*

**DOI:** 10.1038/ncomms11465

**Published:** 2016-04-20

**Authors:** Phelim Bradley, N. Claire Gordon, Timothy M. Walker, Laura Dunn, Simon Heys, Bill Huang, Sarah Earle, Louise J. Pankhurst, Luke Anson, Mariateresa de Cesare, Paolo Piazza, Antonina A. Votintseva, Tanya Golubchik, Daniel J. Wilson, David H. Wyllie, Roland Diel, Stefan Niemann, Silke Feuerriegel, Thomas A. Kohl, Nazir Ismail, Shaheed V. Omar, E. Grace Smith, David Buck, Gil McVean, A. Sarah Walker, Tim E. A. Peto, Derrick W. Crook, Zamin Iqbal

Nature Communications 6: Article number: 10063 10.1038/ncomms10063 (2015); Published: 12212015; Updated: 04202016

In Supplementary Data 3 of this Article, one of the *Staphylococcus aureus* accession codes is incorrect, as follows:

SRR2101499 should be ERR1197981.

