# Supplementary material for: Rapid antibiotic-resistance predictions from genome sequence data for Staphylococcus aureus and Mycobacterium tuberculosis
Source: Nat Commun. 2015 Dec 21;6:10063. doi: 10.1038/ncomms10063 (PMC4703848; doi:10.1038/ncomms10063)
Supplement: Supplementary Information — Supplementary Figures 1-12 and Supplementary Tables 1-15 [file ncomms10063-s1.pdf]

Figures

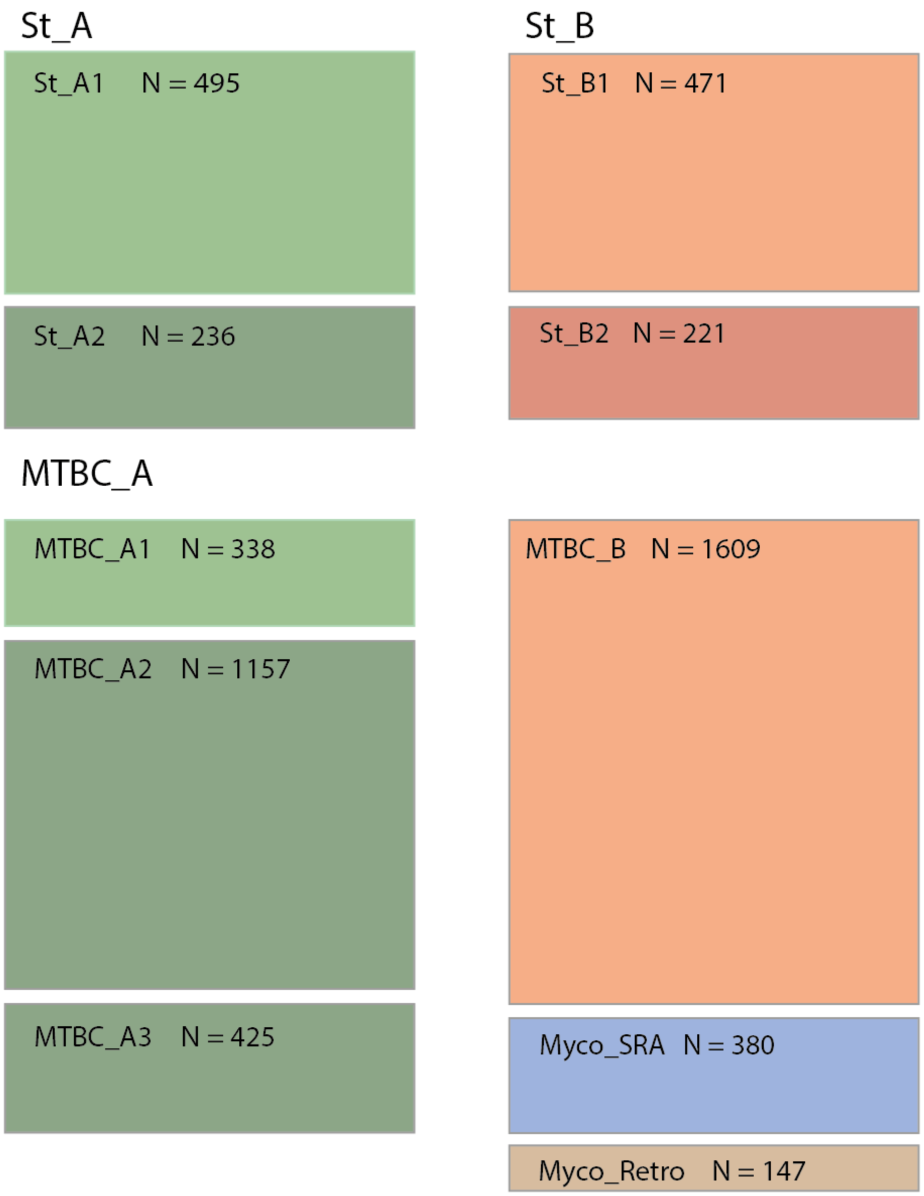

Supplementary Figure 1 Overview of data sets used for training/validation of species-identification and resistance prediction. Left hand column shows datasets used for training, and right-hand column those used for validation. Abbreviations used are St for Staphylococcus, Myco for Mycobacteria, and MTBC for *M. tuberculosis* Complex. See Supplementary Table 1 for details on what phenotype and metadata these datasets each have.
S. aureus species training: St_A = St_A1 + St_A2,
S. aureus species validation: St_B1 + St_B2,
S. aureus resistance training: St_A1,
S. aureus resistance validation: St_B1.
M. tuberculosis species training: MTBC_A1 + Myco_SRA,
M. tuberculosis species validation: MTBC_A2 + Myco_Retro,

M. tuberculosis resistance training: MTBC_A1 + MTBC_A2 + MTBC_A3,
M. tuberculosis resistance validation: MTBC_B.

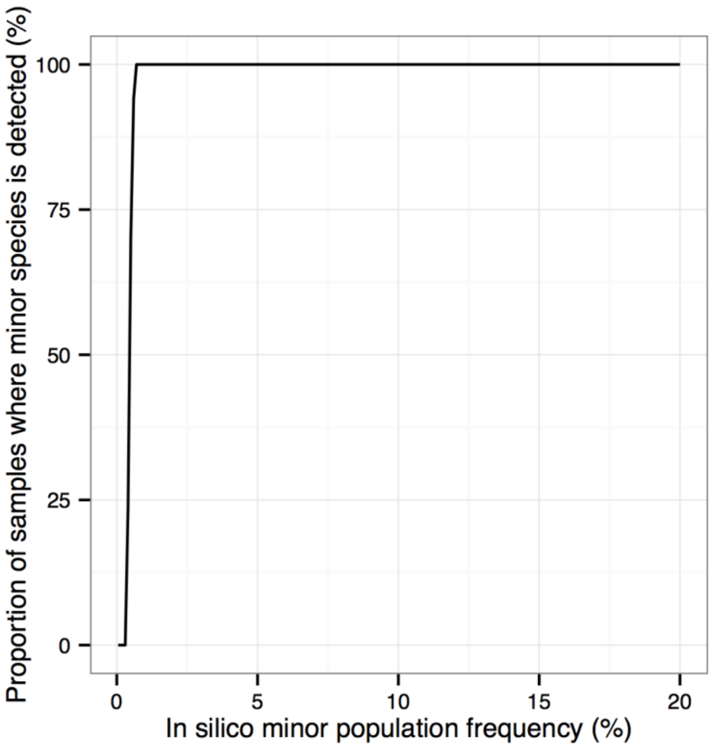

Supplementary Figure 2. Power to detect mixed *S. aureus* and Coagulase-negative *Staphylococcus* (*S. epidermidis and S. haemolyticus*) (Simulation 1). 540 *in silico* mixtures of *S. epidermidis/S. aureus* and *S. haemolyticus/S. aureus* were created at different mixture proportions (see Methods).

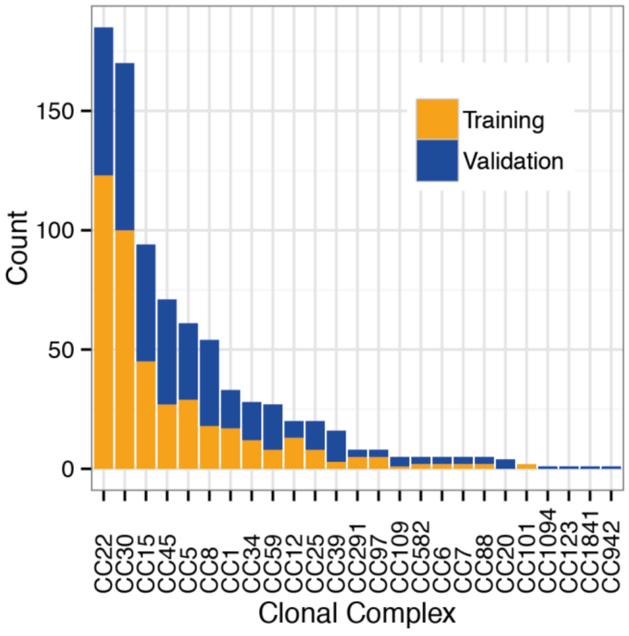

Supplementary Figure 3. Counts of each clonal complex in *S. aureus* training set St_A1 and validation set St_B1.

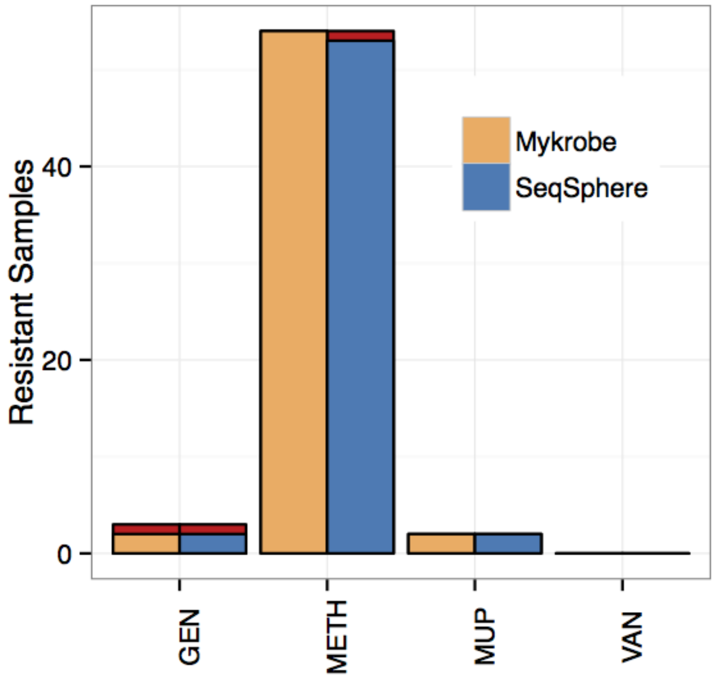

Supplementary Figure 4: Proportion of resistant samples of *S. aureus* in dataset St_B2 correctly identified as resistant by *Mykrobe predictor* (yellow) and SeqSphere (blue), as compared with consensus phenotype - false negatives in red.

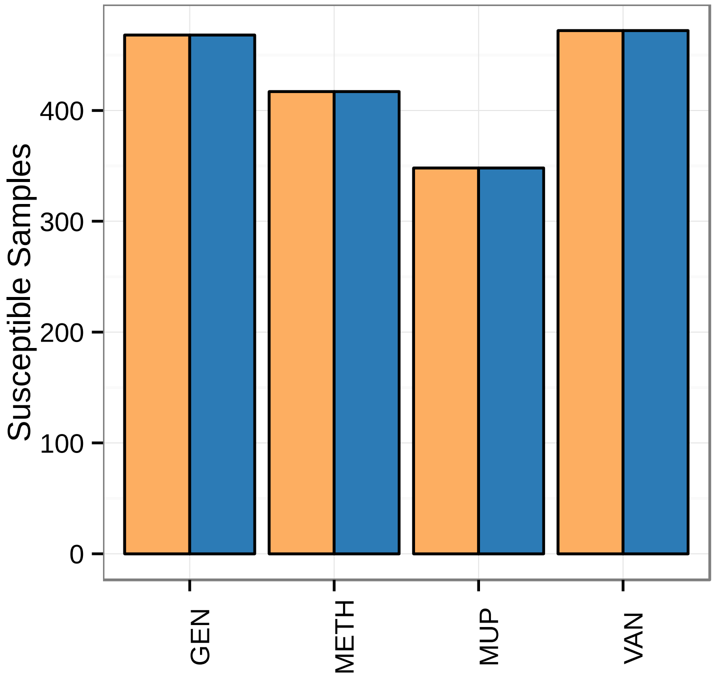

Supplementary Figure 5: Proportion of resistant samples of *S. aureus* in dataset St_B2 correctly identified as susceptible by *Mykrobe predictor* (yellow) and SeqSphere (blue) compared with consensus phenotype - false positives in red.

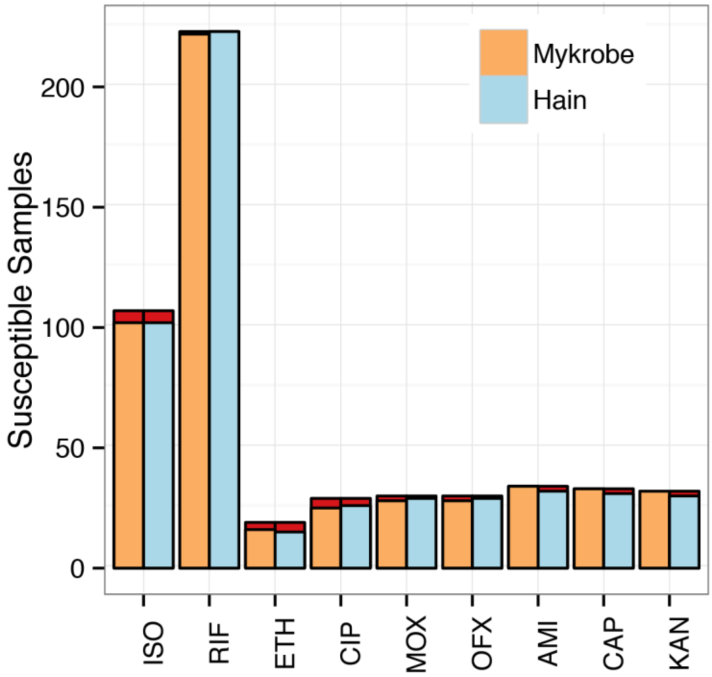

Supplementary Figure 6 Proportion of phenotypically susceptible samples correctly identified as susceptible by *Mykrobe predictor* (yellow) and Hain assay (blue), on dataset MTBC_A1. Height of bars gives total susceptible samples, and false positive calls are shaded red.

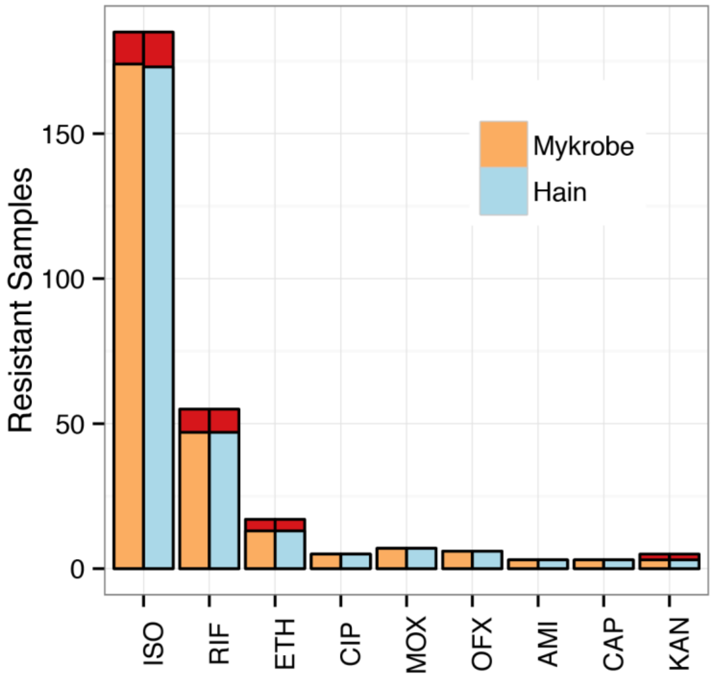

Supplementary Figure 7: Proportion of phenotypically resistant samples correctly identified as resistant by *Mykrobe predictor* and Hain assay, on dataset MTBC_A1. Height of bars gives total resistant samples, and false negative calls are shaded red

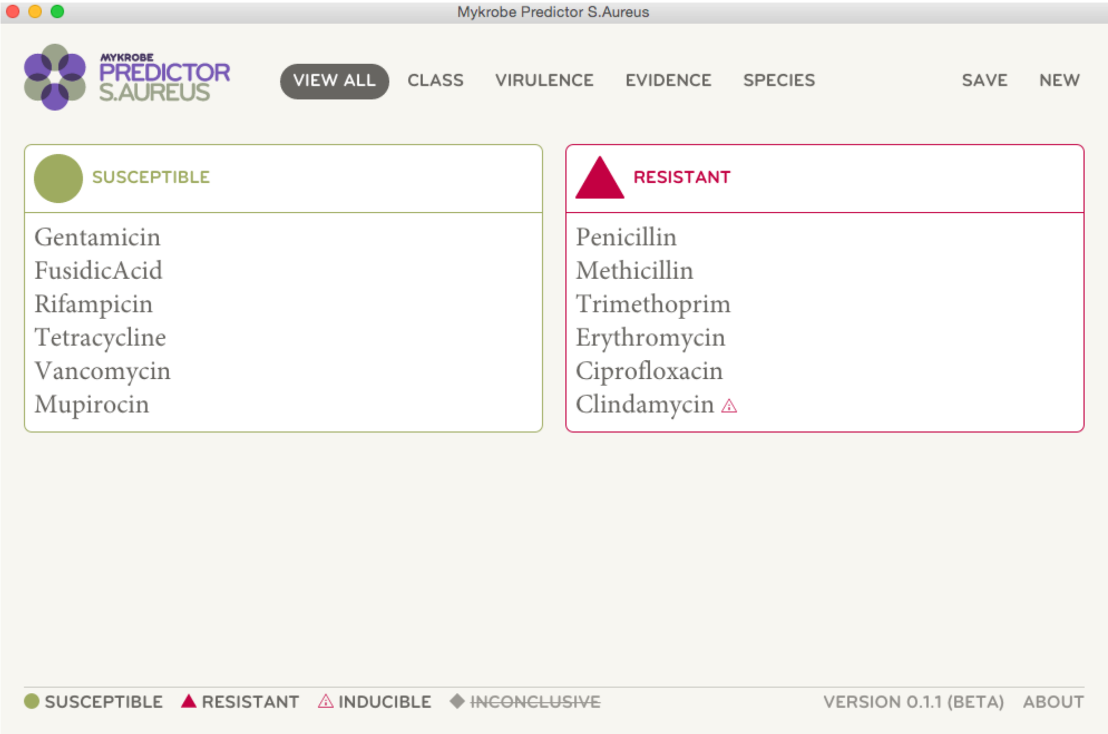

Supplementary Figure 8: Screenshot of the *Mykrobe predictor S. aureus* desktop app showing drugs split by resistant or susceptible prediction.

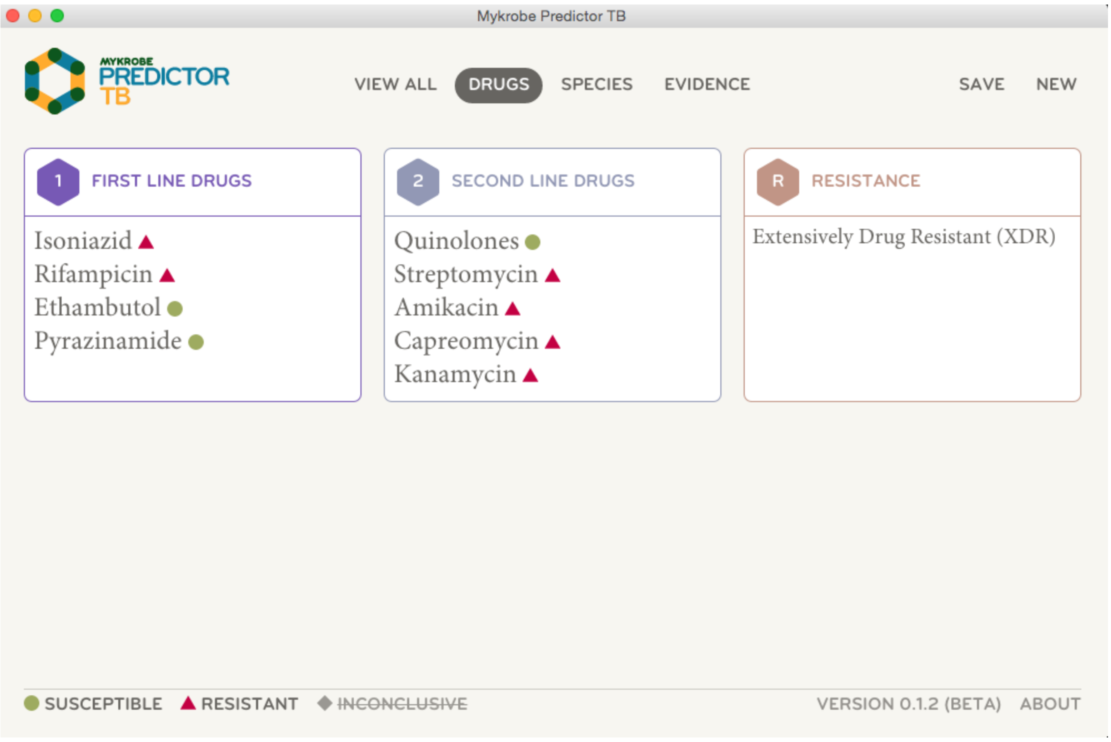

Supplementary Figure 9: Screenshot of the *Mykrobe predictor* TB desktop app showing drugs split by first and second line (TB) alongside resistant or susceptible prediction.

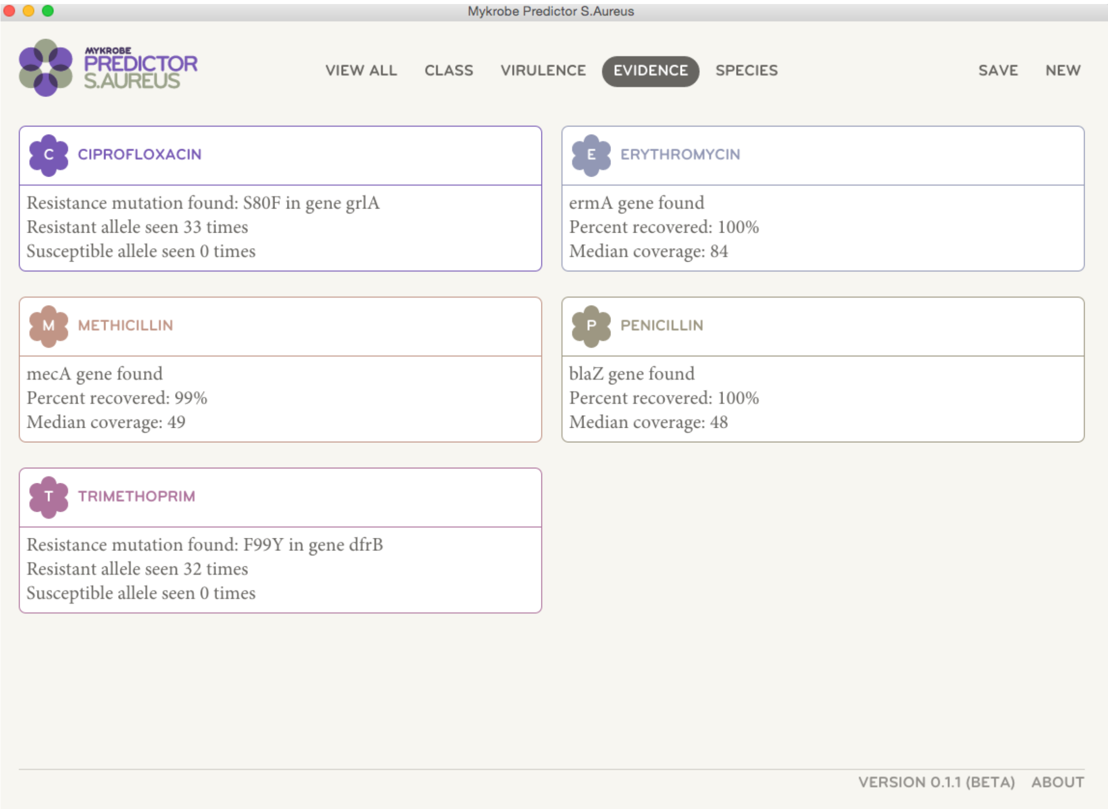

Supplementary Figure 10: Screenshot of the *Mykrobe predictor S. aureus* desktop app with evidence for each of the resistance calls.

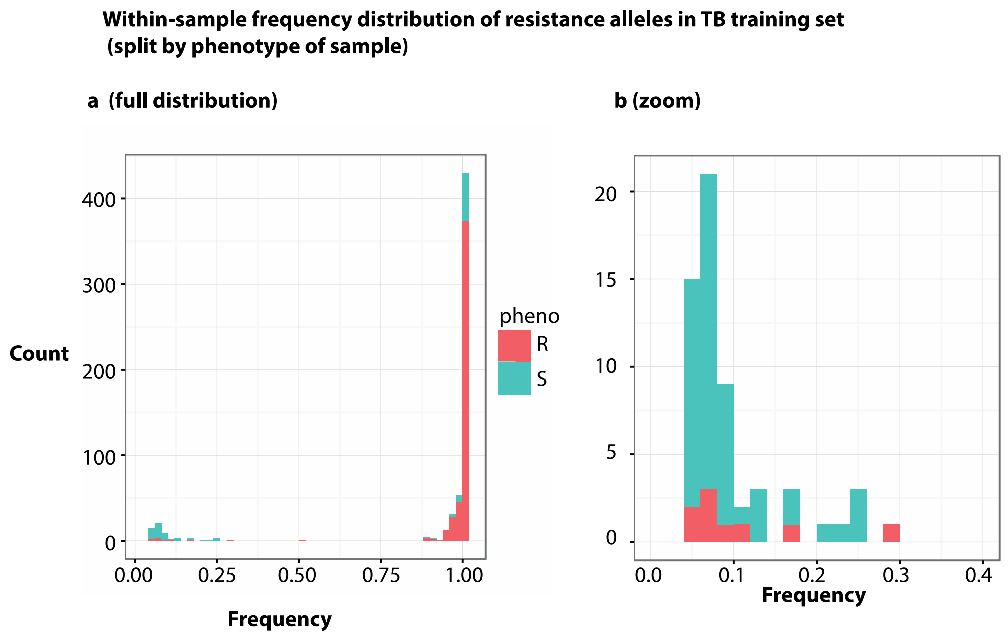

Supplementary Figure 11:
Within-sample frequency of resistant alleles in the training set MTBC_A1 coloured by associated phenotype. Alleles at frequency > 90% with an associated Susceptible phenotype are as follows (where X means any amino acid):
embB M306X: 31, *fabG1* C-15X: 5, *fabG1* G-17X: 1, *fabG1* T-8X: 2, *gyrA* A90X: 3, *gyrA* D94X: 9, *gyrA* S91X: 2, *katG* S315X : 1, *rpoB* D435X: 3, *rpoB* H445X: 3, *rpoB* L430X: 1, *rpoB* L452X : 3, *rpoB* S450X: 2, rrs *G1484X* : 1. The dominant mutations, *embB* M306V and M306I, are a known phenomenon, as the Minimum Inhibitory Concentration (MIC) of resistance caused by these mutations are very close to the critical concentration – causing stochastic “flip-flopping” of the test, depending on whether resistance is just above or below the threshold. This is an artifact of forcing a binary classification on a quantitative trait. The *rpoB* mutations are discussed in the results.

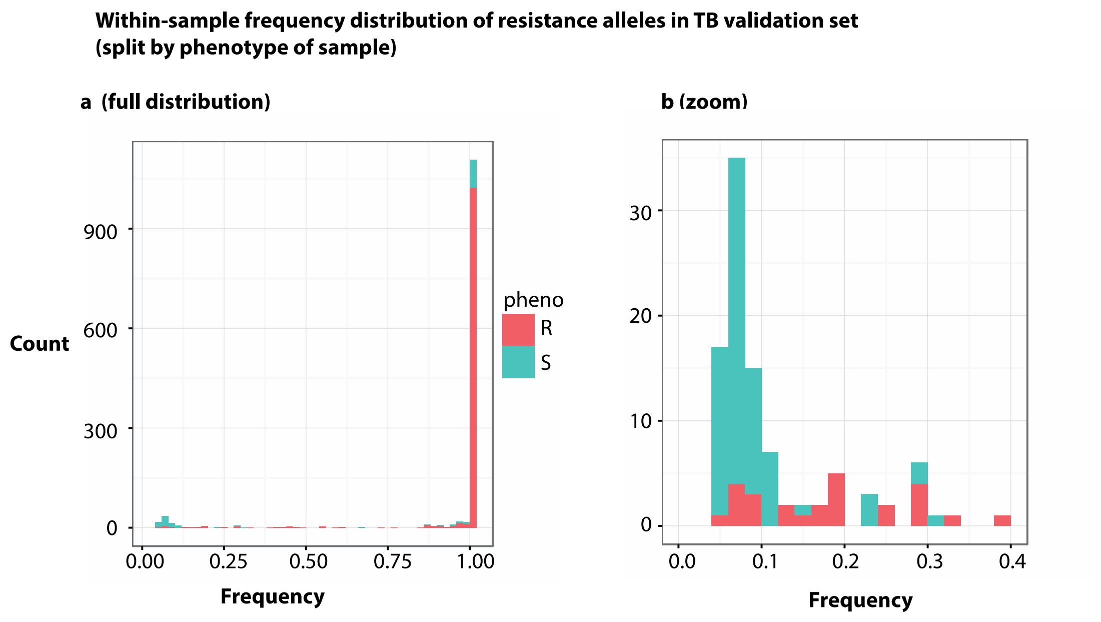

Supplementary Figure 12:

Within-sample frequency of resistant allele in validation set MTBC_B coloured by associated phenotype. Alleles at frequency > 90% with an associated Susceptible phenotype are:

embB M306X: 54, *fabG1* C-15X: 9, *katG* S315X: 8, *rpoB* L430X: 3, *rpoB* L452X: 3, *rpoB* Q429X : 1, *rpoB* Q432X: 1, *rpoB* S450X: 4, *rpsL* K43R: 6, rrs *A1401X* : 7, rrs *C1402X* : 1, rrs *C517X*: 1

Tables

Set	Species truth	Phenotype information	Supplementary Data File
St_A1	Mapping to reference	Vitek or Disc	1
St_A2	Mapping to reference or SRA metadata	None	2
St_B1	Mapping to reference	Disc+Phoenix (+nitrocefin+Etest for consensus)	3
St_B2	Mapping to reference or SRA metadata	None	4
St_PVL		PCR for PVL	5
MTBC_A1	Hain	Traditional DST	6
MTBC_A2	Hain	Traditional DST	7
MTBC_A3	None	Traditional DST	8
MTBC_B	None	Traditional DST	9
MTBC_SRA	SRA metadata	None	10
Myc_retro	PCR	None	11

Supplementary Table 1: Summary of metadata for each dataset used. See Supplementary Figure 1 for how these datasets were combined for different analyses.

Drug	Total	FN(R)	FP(S)	VME	ME	PPV	NPV
PEN	495	3 (437)	2 (58)	0.7% (0.1%-2.0%)	3.4% (0.4%-11.9%)	99.5% (98.4%-99.9%)	94.9% (85.9%-98.9%)
CIP	495	6 (170)	8 (325)	3.5% (1.3%-7.5%)	2.5% (1.1%-4.8%)	95.3% (91.0%-98.0%)	98.1% (96.0%-99.3%)
METH	495	0 (158)	2 (337)	0.0% (0%-2.3%)	0.6% (0.1%-2.1%)	98.8% (95.6%-99.8%)	100.0% (98.9%-100%)
ERY	495	0 (133)	1 (362)	0.0% (0%-2.7%)	0.3% (0.0%-1.5%)	99.3% (95.9%-100.0%)	100.0% (99.0%-100%)
CLIN	177	0 (88)	1 (89)	0.0% (0%-4.1%)	1.1% (0.0%-6.1%)	98.9% (93.9%-100.0%)	100.0% (95.9%-100%)
FUS	495	0 (39)	4 (456)	0.0% (0%-9.0%)	0.9% (0.2%-2.2%)	90.7% (77.9%-97.4%)	100.0% (99.2%-100%)
TET	495	0 (27)	0 (468)	0.0% (0%-12.8%)	0.0% (0%-0.8%)	100.0% (87.2%-100%)	100.0% (99.2%-100%)
TRIM	317	3 (13)	0 (304)	23.1% (5.0%-53.8%)	0.0% (0%-1.2%)	100.0% (69.2%-100%)	99.0% (97.2%-99.8%)
GEN	495	0 (7)	0 (488)	N/A	0.0% (0%-0.8%)	100.0% (59.0%-100%)	100.0% (99.2%-100%)
RIF	495	0 (2)	2 (493)	N/A	0.4% (0.0%-1.5%)	50.0% (6.8%-93.2%)	100.0% (99.3%-100%)

MUP	178	0 (2)	2 (176)	N/A	1.1% (0.1%-4.0%)	50.0% (6.8%-93.2%)	100.0% (97.9%-100%)
VAN	495	0 (0)	0 (495)	N/A	0.0% (0%-0.7%)	100% (100%-100%)	100.0% (99.3%-100%)

Supplementary Table 2 Results for *Mykrobe predictor* on the Staphylococcal training set (St_A1). Resistance prediction results for *Mykrobe predictor* on the *S. aureus* training set St_A1 treating Stokes Disc test as truth. FN: False negative calls. R: total number of resistant samples. FP: false positives. S: total number of susceptible samples. VME: very major error rate (false negative rate. ME: major error rate (false positive rate). PPV: positive predictive value. NPV: negative predictive value. N/A: Not Applicable. Error rates shown with 95% CI calculated by Clopper-Pearson; FN/FP rate only shown where number of resistant/susceptible samples >10.

Drug	Total	FN(R)	FP(S)	VME	ME	PPV	NPV
PEN	470	28 (377)	14 (93)	7.4% (5.0%-10.6%)	15.1% (8.5%-24.0%)	96.1% (93.6%-97.9%)	73.8% (64.4%-81.9%)
ERY	471	2 (79)	6 (392)	2.5% (0.3%-8.8%)	1.5% (0.6%-3.3%)	92.8% (84.9%-97.3%)	99.5% (98.2%-99.9%)
CIP	471	1 (65)	7 (406)	1.5% (0.0%-8.3%)	1.7% (0.7%-3.5%)	90.1% (80.7%-95.9%)	99.8% (98.6%-100.0%)
METH	471	0 (54)	2 (417)	0.0% (0%-6.6%)	0.5% (0.1%-1.7%)	96.4% (87.7%-99.6%)	100.0% (99.1%-100%)
FUS	470	0 (40)	1 (430)	0.0% (0%-8.8%)	0.2% (0.0%-1.3%)	97.6% (87.1%-99.9%)	100.0% (99.1%-100%)
CLIN	117	2 (21)	0 (96)	9.5% (1.2%-30.4%)	0.0% (0%-3.8%)	100.0% (82.4%-100%)	98.0% (92.8%-99.8%)
TET	471	0 (17)	1 (454)	0.0% (0%-19.5%)	0.2% (0.0%-1.2%)	94.4% (72.7%-99.9%)	100.0% (99.2%-100%)
RIF	471	1 (5)	0 (466)	N/A	0.0% (0%-0.8%)	100.0% (39.8%-100%)	99.8% (98.8%-100.0%)
GEN	471	1 (3)	0 (468)	N/A	0.0% (0%-0.8%)	100.0% (15.8%-100%)	99.8% (98.8%-100.0%)
MUP	350	0 (2)	0 (348)	N/A	0.0% (0%-1.1%)	100.0% (15.8%-100%)	100.0% (98.9%-100%)
VAN	472	0 (0)	0 (472)	N/A	0.0% (0%-0.8%)	100% (100%-100%)	100.0% (99.2%-100%)

Supplementary Table 3: Validation (St_B1) *S. aureus* Disc results. Resistance prediction results for BSAC Disc test on the *S. aureus* validation set St_B1 compared against the consensus phenotype. FN: False negative calls. R: total

number of resistant samples. FP: false positives. S: total number of susceptible samples. VME: very major error rate (false negative rate. ME: major error rate (false positive rate). PPV: positive predictive value. NPV: negative predictive value. N/A: Not Applicable. Error rates shown with 95% CI calculated by Clopper-Pearson; FN/FP rate only shown where number of resistant/susceptible samples >10

Drug	Total	FN(R)	FP(S)	VME	ME	PPV	NPV
PEN	471	6 (377)	15 (94)	1.6% (0.6%-3.4%)	16.0% (9.2%-25.0%)	96.1% (93.7%-97.8%)	92.9% (85.3%-97.4%)
ERY	470	1 (79)	0 (391)	1.3% (0.0%-6.9%)	0.0% (0%-0.9%)	100.0% (95.4%-100%)	99.7% (98.6%-100.0%)
CIP	471	7 (65)	0 (406)	10.8% (4.4%-20.9%)	0.0% (0%-0.9%)	100.0% (93.8%-100%)	98.3% (96.5%-99.3%)
METH	471	2 (54)	79 (417)	3.7% (0.5%-12.7%)	18.9% (15.3%-23.0%)	39.7% (31.3%-48.6%)	99.4% (97.9%-99.9%)
FUS	471	2 (41)	1 (430)	4.9% (0.6%-16.5%)	0.2% (0.0%-1.3%)	97.5% (86.8%-99.9%)	99.5% (98.3%-99.9%)
CLIN	122	7 (25)	1 (97)	28.0% (12.1%-49.4%)	1.0% (0.0%-5.6%)	94.7% (74.0%-99.9%)	93.2% (86.5%-97.2%)
TET	471	0 (17)	2 (454)	0.0% (0%-19.5%)	0.4% (0.1%-1.6%)	89.5% (66.9%-98.7%)	100.0% (99.2%-100%)
RIF	471	0 (5)	0 (466)	N/A	0.0% (0%-0.8%)	100.0% (47.8%-100%)	100.0% (99.2%-100%)
GEN	471	1 (3)	2 (468)	N/A	0.4% (0.1%-1.5%)	50.0% (6.8%-93.2%)	99.8% (98.8%-100.0%)
MUP	350	0 (2)	0 (348)	N/A	0.0% (0%-1.1%)	100.0% (15.8%-100%)	100.0% (98.9%-100%)
VAN	472	0 (0)	0 (472)	N/A	0.0% (0%-0.8%)	100% (100%-100%)	100.0% (99.2%-100%)

Supplementary Table 4: Validation (St_B1) *S. aureus* Phoenix Results. Resistance prediction results for BD Phoenix test on the *S. aureus* validation set St_B1 compared against the consensus phenotype. FN: False negative calls. R: total number of resistant samples. FP: false positives. S: total number of susceptible samples. VME: very major error rate (false negative rate. ME: major error rate (false positive rate). PPV: positive predictive value. NPV: negative predictive value. N/A: Not Applicable. Error rates shown with 95% CI calculated by Clopper-Pearson; FN/FP rate only shown where number of resistant/susceptible samples >10

Drug	Total	FN(R)	FP(S)	VME	ME	PPV	NPV
ERY	471	1 (79)	392 (392)	1.3% (0.0%-6.9%)	100.0% (99.1%-0%)	16.6% (13.3%-20.3%)	0.0% (100%-97.5%)
METH	471	1 (54)	0 (417)	1.9% (0.0%-9.9%)	0.0% (0%-0.9%)	100.0% (93.3%-100%)	99.8% (98.7%-100.0%)

CLIN	122	0 (25)	97 (97)	0.0% (0%-13.7%)	100.0% (96.3%-0%)	20.5% (13.7%-28.7%)	100% (100%-100%)
GEN	471	1 (3)	0 (468)	N/A	0.0% (0%-0.8%)	100.0% (15.8%-100%)	99.8% (98.8%-100.0%)
MUP	350	0 (2)	0 (348)	N/A	0.0% (0%-1.1%)	100.0% (15.8%-100%)	100.0% (98.9%-100%)
VAN	472	0 (0)	0 (472)	N/A	0.0% (0%-0.8%)	100% (100%-100%)	100.0% (99.2%-100%)

Supplementary Table 5 Validation (St_B1) *S. aureus* SeqSphere results. Resistance prediction results for SeqSphere on the *S. aureus* validation set St_B1 compared against the consensus phenotype. FN: False negative calls. R: total number of resistant samples. FP: false positives. S: total number of susceptible samples. VME: very major error rate (false negative rate. ME: major error rate (false positive rate). PPV: positive predictive value. NPV: negative predictive value. N/A: Not Applicable. Error rates shown with 95% CI calculated by Clopper-Pearson.

	Predict																	
		tub	bov	afr	MTBC	avi	mar	ulc	int	abs	for	gor	mal	xen	kan	szu	sme	che	NTM
	tub	1192	0	1	2	0	0	0	0	0	0	0	0	0	0	0	0	0	0
	bov	0	7	0	0	0	0	0	0	0	0	0	0	0	0	0	0	0	0
	afr	0	0	0	4	0	0	0	0	0	0	0	0	0	0	0	0	0	0
	MTBC	10	1	0	0	0	0	0	0	0	0	0	0	0	0	0	0	0	0
	avi	0	0	0	0	26	0	0	1	0	0	0	0	0	0	0	0	0	0
	mar	0	0	0	0	0	0	2	0	0	0	0	0	0	0	0	0	0	0
	ulc	0	0	0	0	0	0	0	0	0	0	0	0	0	0	0	0	0	0
	int	0	0	0	0	1	0	0	19	1	0	0	0	0	0	0	0	0	0
	abs	0	0	0	0	0	0	0	0	15	0	0	0	0	0	0	0	0	0
	for	0	0	0	0	0	0	0	0	0	3	0	0	0	0	0	0	0	0
	gor	0	0	0	0	0	0	0	0	0	0	0	0	0	0	0	2	0	4
	mal	0	0	0	0	0	0	0	0	0	0	0	2	0	0	0	0	0	0
	xen	0	0	0	0	0	0	0	0	0	0	0	0	2	0	0	0	0	0
	kan	0	0	0	0	1	0	0	0	0	0	0	0	0	1	0	0	0	0
	szu	0	0	0	0	0	0	0	0	0	0	0	0	0	0	1	1	0	0
	sme	0	0	0	0	0	0	0	0	0	0	0	0	0	0	0	0	0	0
	che	0	0	0	0	0	0	0	0	2	0	0	0	0	0	0	0	0	0
Truth	NTM	0	0	0	0	0	0	0	0	0	0	0	0	0	0	0	0	0	0

Supplementary Table 6 Extended Mycobacterium Species confusion matrix on validation set (MTBC_A2 + Myco_Retro). tub - M. tuberculosis, bov - M. bovis, afr - M. africanum, MTBC - Mycobacterium Tuberculosis Complex, avi - M. avium, mar -

M. marinum, ulc - M. ulcerans, int - M. intracellulare, abs - M. abscessus, for - M. fortuitum, gor - M. gordonae, mal - M. malmoense, xen - M. xenopi, kan - M. kansasii, szu - M. szulgae, sme - M. smegmatis, che - M. chelonae, NTM – Non Tuberculous Mycobacteria.

Drug	Total	FN(R)	FP(S)	VME	ME	PPV	NPV
ISO	1911	33 (275)	11 (1636)	12.0% (8.4%-16.4%)	0.7% (0.3%-1.2%)	95.7% (92.4%-97.8%)	98.0% (97.2%-98.6%)
RIF	1870	11 (102)	15 (1768)	10.8% (5.5%-18.5%)	0.8% (0.5%-1.4%)	85.8% (77.7%-91.9%)	99.4% (98.9%-99.7%)
STREP	477	32 (73)	0 (404)	43.8% (32.2%-55.9%)	0.0% (0%-0.9%)	100.0% (91.4%-0%)	92.7% (89.8%-94.9%)
ETH	1889	19 (57)	35 (1832)	33.3% (21.4%-47.1%)	1.9% (1.3%-2.6%)	52.1% (40.0%-63.9%)	99.0% (98.4%-99.4%)
CIP	274	4 (22)	6 (252)	18.2% (5.2%-40.3%)	2.4% (0.9%-5.1%)	75.0% (53.3%-90.2%)	98.4% (96.0%-99.6%)
OFX	130	1 (16)	4 (114)	6.3% (0.2%-30.2%)	3.5% (1.0%-8.7%)	78.9% (54.4%-93.9%)	99.1% (95.1%-100.0%)
MOX	131	0 (15)	5 (116)	0.0% (0%-21.8%)	4.3% (1.4%-9.8%)	75.0% (50.9%-91.3%)	100.0% (96.7%-0%)
KAN	104	4 (9)	1 (95)	N/A	1.1% (0.0%-5.7%)	83.3% (35.9%-99.6%)	95.9% (89.9%-98.9%)
CAP	106	2 (7)	2 (99)	N/A	2.0% (0.2%-7.1%)	71.4% (29.0%-96.3%)	98.0% (92.9%-99.8%)
AMI	112	0 (6)	1 (106)	N/A	0.9% (0.0%-5.1%)	85.7% (42.1%-99.6%)	100.0% (96.5%-0%)

Supplementary Table 7 Results for *Mykrobe predictor* on the *M. tuberculosis* training set (MTBC_A1). Resistance prediction results for *Mykrobe predictor* on the *M. tuberculosis* training set MTBC_A1 compared against the consensus phenotype. FN: False negative calls. R: total number of resistant samples. FP: false positives. S: total number of susceptible samples. VME: very major error rate (false negative rate. ME: major error rate (false positive rate). PPV: positive predictive value. NPV: negative predictive value. N/A: Not Applicable. Error rates shown with 95% CI calculated by Clopper-Pearson; FN/FP rate only shown where number of resistant/susceptible samples >10

Drug	Total	FN(R)	FP(S)	VME	ME	PPV	NPV
ISO	1911	39 (275)	9 (1636)	14.2% (10.3%-18.9%)	0.6% (0.3%-1.0%)	96.3% (93.1%-98.3%)	97.7% (96.8%-98.3%)
RIF	1870	10 (101)	13 (1769)	9.9% (4.9%-17.5%)	0.7% (0.4%-1.3%)	87.5% (79.6%-93.2%)	99.4% (99.0%-99.7%)
STREP	477	31 (73)	13 (404)	42.5% (31.0%-54.6%)	3.2% (1.7%-5.4%)	76.4% (63.0%-86.8%)	92.7% (89.7%-95.0%)
ETH	18	13	41	23.2% (13.0%-	2.2% (1.6%-	51.2% (40.0%-	99.3% (98.8%-

	89	(56)	(1833)	36.4%)	3.0%)	62.3%)	99.6%)
CIP	274	2 (22)	10 (252)	9.1% (1.1%-29.2%)	4.0% (1.9%-7.2%)	66.7% (47.2%-82.7%)	99.2% (97.1%-99.9%)
MOX	131	0 (15)	5 (116)	0.0% (0%-21.8%)	4.3% (1.4%-9.8%)	75.0% (50.9%-91.3%)	100.0% (96.7%-100%)
KAN	104	4 (9)	0 (95)	N/A	0.0% (0%-3.8%)	100.0% (47.8%-100%)	96.0% (90.0%-98.9%)
CAP	106	2 (7)	0 (99)	N/A	0.0% (0%-3.7%)	100.0% (47.8%-100%)	98.0% (93.0%-99.8%)
AMI	112	1 (6)	0 (106)	N/A	0.0% (0%-3.4%)	100.0% (47.8%-100%)	99.1% (94.9%-100.0%)

Supplementary Table 8 Results for *KvarQ* on the *M. tuberculosis* training set (MTBC_A1). Resistance prediction results for *KvarQ* on the *M. tuberculosis* training set MTBC_A1 compared against the consensus phenotype. FN: False negative calls. R: total number of resistant samples. FP: false positives. S: total number of susceptible samples. VME: very major error rate (false negative rate. ME: major error rate (false positive rate). PPV: positive predictive value. NPV: negative predictive value. N/A: Not Applicable. Error rates shown with 95% CI calculated by Clopper-Pearson; FN/FP rate only shown where number of resistant/susceptible samples >10

Drug	Total	FN(R)	FP(S)	VME	ME	PPV	NPV
ISO	1586	58 (370)	17 (1216)	15.7% (12.1%-19.8%)	1.4% (0.8%-2.2%)	94.8% (91.9%-97.0%)	95.4% (94.1%-96.5%)
STREP	1580	75 (353)	9 (1227)	21.2% (17.1%-25.9%)	0.7% (0.3%-1.4%)	96.9% (94.1%-98.6%)	94.2% (92.8%-95.4%)
RIF	1565	19 (303)	12 (1262)	6.3% (3.8%-9.6%)	1.0% (0.5%-1.7%)	95.9% (93.0%-97.9%)	98.5% (97.7%-99.1%)
ETH	1585	55 (194)	59 (1391)	28.4% (22.1%-35.2%)	4.2% (3.2%-5.4%)	70.2% (63.3%-76.5%)	96.0% (94.9%-97.0%)
AMI	682	6 (59)	6 (623)	10.2% (3.8%-20.8%)	1.0% (0.4%-2.1%)	89.8% (79.2%-96.2%)	99.0% (97.9%-99.6%)
CAP	685	9 (55)	13 (630)	16.4% (7.8%-28.8%)	2.1% (1.1%-3.5%)	78.0% (65.3%-87.7%)	98.6% (97.3%-99.3%)
OFX	706	5 (13)	0 (693)	38.5% (13.9%-68.4%)	0.0% (0%-0.5%)	100.0% (63.1%-100%)	99.3% (98.3%-99.8%)
MOX	487	4 (9)	0 (478)	N/A	0.0% (0%-0.8%)	100.0% (47.8%-100%)	99.2% (97.9%-99.8%)
KAN	469	6 (9)	2 (460)	N/A	0.4% (0.1%-1.6%)	60.0% (14.7%-94.7%)	98.7% (97.2%-99.5%)
CI	1	0 (0)	0 (1)	N/A	0.0% (0%-	100% (100%-	100.0% (2.5%-100%)

P					97.5%)	100%)	

Supplementary Table 9 Results for *Mykrobe predictor* on the *M. tuberculosis* validation set (MTBC_B). Resistance prediction results for *Mykrobe predictor* on the *M. tuberculosis* validation set MTBC_B compared against the consensus phenotype. FN: False negative calls. R: total number of resistant samples. FP: false positives. S: total number of susceptible samples. VME: very major error rate (false negative rate. ME: major error rate (false positive rate). PPV: positive predictive value. NPV: negative predictive value. N/A: Not Applicable. Error rates shown with 95% CI calculated by Clopper-Pearson; FN/FP rate only shown where number of resistant/susceptible samples >10

Drug	Total	FN(R)	FP(S)	VME	ME	PPV	NPV
ISO	1586	62 (370)	17 (1216)	16.8% (13.1%-21.0%)	1.4% (0.8%-2.2%)	94.8% (91.8%-96.9%)	95.1% (93.7%-96.2%)
STREP	1580	72 (353)	87 (1227)	20.4% (16.3%-25.0%)	7.1% (5.7%-8.7%)	76.4% (71.7%-80.6%)	94.1% (92.6%-95.3%)
RIF	1565	28 (303)	12 (1262)	9.2% (6.2%-13.1%)	1.0% (0.5%-1.7%)	95.8% (92.8%-97.8%)	97.8% (96.8%-98.5%)
ETH	1585	46 (194)	62 (1391)	23.7% (17.9%-30.3%)	4.5% (3.4%-5.7%)	70.5% (63.8%-76.6%)	96.7% (95.6%-97.5%)
AMI	682	15 (59)	2 (623)	25.4% (15.0%-38.4%)	0.3% (0.0%-1.2%)	95.7% (85.2%-99.5%)	97.6% (96.1%-98.7%)
CAP	685	16 (55)	8 (630)	29.1% (17.6%-42.9%)	1.3% (0.5%-2.5%)	83.0% (69.2%-92.4%)	97.5% (96.0%-98.6%)
MOX	487	4 (9)	1 (478)	N/A	0.2% (0.0%-1.2%)	83.3% (35.9%-99.6%)	99.2% (97.9%-99.8%)
KAN	469	6 (9)	0 (460)	N/A	0.0% (0%-0.8%)	100.0% (29.2%-100%)	98.7% (97.2%-99.5%)
CIP	1	0 (0)	0 (1)	N/A	0.0% (0%-97.5%)	100% (100%-100%)	100.0% (2.5%-100%)

Supplementary Table 10 Results for *KvarQ* on the *M. tuberculosis* validation set (MTBC_B). Resistance prediction results for *KvarQ* on the *M. tuberculosis* validation set MTBC_B compared against the consensus phenotype. FN: False negative calls. R: total number of resistant samples. FP: false positives. S: total number of susceptible samples. VME: very major error rate (false negative rate. ME: major error rate (false positive rate). PPV: positive predictive value. NPV: negative predictive value. N/A: Not Applicable. Error rates shown with 95% CI calculated by Clopper-Pearson.

katA_1 gi|29165615:1332093-1333616 Staphylococcus aureus subsp. aureus N315 chromosome, complete genome
katA_2 GCA_000236925.1
katA_3 actually katB AY702101.2 Staphylococcus xylosus catalase B (katB) gene, complete cds

SA21310_0235:EGL89674 EGL89674-1 exon:ANNOTATED_protein_coding
contig00008 dna:supercontig supercontig:GCA_000215425.2:contig00008:106000:107517:-1
SACIG1233_1932:EHT60300 EHT60300-1 exon:ANNOTATED_protein_coding
PSABG_19_CIG1233.contig.11_1 dna:supercontig supercontig:GCA_000248775.2:PSABG_19_CIG1233.contig.11_1:836836:838353:1
MRGR3_1037:EOR40753 EOR40753-1 exon:ANNOTATED_protein_coding
MRGR3_28 dna:supercontig supercontig:GCA_000401475.1:MRGR3_28:60970:62493:-1
SACIG1835_1413:EHT44120 EHT44120-1 exon:ANNOTATED_protein_coding
PSABG_14_CIG1835.contig.7_1 dna:supercontig supercontig:GCA_000248655.2:PSABG_14_CIG1835.contig.7_1:791377:792894:1
SA957_1228:AGW33725 AGW33725-1 exon:ANNOTATED_protein_coding
Chromosome dna:chromosome chromosome:GCA_000470845.1:Chromosome:1340542:1342059:1
SPSE_2462:ADX77670 ADX77670-1 exon:ANNOTATED_protein_coding
Chromosome dna:chromosome chromosome:GCA_000189495.1:Chromosome:2535659:2537650:1
SOJ_00740:EJX19135 EJX19135-1 exon:ANNOTATED_protein_coding
155.SOJ.1_1 dna:supercontig supercontig:GCA_000294465.1:155.SOJ.1_1:71651:73642:1
SH1573:BAE04882 BAE04882-1 exon:ANNOTATED_protein_coding
Chromosome dna:chromosome chromosome:GCA_000009865.1:Chromosome:1619847:1621358:-1
SH1573:BAE04882 BAE04882-1 exon:ANNOTATED_protein_coding
Chromosome dna:chromosome chromosome:GCA_000009865.1:Chromosome:1619847:1621358:-1
CR01_140002:CDI71796 CDI71796-1 exon:ANNOTATED_protein_coding
CR01 dna:supercontig supercontig:GCA_000499705.1:CR01:1653894:1655405:-1
SPSE_0142:ADX75489 ADX75489-1 exon:ANNOTATED_protein_coding
Chromosome dna:chromosome chromosome:GCA_000189495.1:Chromosome:157630:159120:1
katA Staphylococcus epidermidis strain DSM1798 catalase (katA) gene, partial cds
katA Staphylococcus epidermidis strain DSM20044 catalase (katA) gene, partial cds
katA Staphylococcus saprophyticus strain GB1 catalase (katA) gene, partial cds
katA Staphylococcus saprophyticus strain DSM20229BK catalase (katA) gene, partial cds
katA Staphylococcus hominis strain DSM20328 catalase (katA) gene, partial cds
katA Staphylococcus haemolyticus strain DSM20263 catalase (katA) gene, partial cds
katA Staphylococcus lugdunensis strain NBL01 catalase (katA) pseudogene, partial sequence
katA Staphylococcus capitis subsp. capitis strain DSM20326 catalase (katA) gene, partial cds

Supplementary Table 11 Accession IDs for alleles/versions of the *katA* gene used to detect presence of any Staphylococcus.

drug	gene	alphabet	pos	ref	alt
TRIM	dfrB	PROTEIN	21	L	V
TRIM	dfrB	PROTEIN	31	H	N
TRIM	dfrB	PROTEIN	41	L	F
TRIM	dfrB	PROTEIN	60	N	I
TRIM	dfrB	PROTEIN	99	F	Y
TRIM	dfrB	PROTEIN	99	F	S
TRIM	dfrB	PROTEIN	99	F	I

TRIM	dfrB	PROTEIN	150	H	R
FUC	fusA	PROTEIN	652	F	S
FUC	fusA	PROTEIN	654	Y	N
FUC	fusA	PROTEIN	456	L	F
FUC	fusA	PROTEIN	461	L	F
FUC	fusA	PROTEIN	326	T	I
FUC	fusA	PROTEIN	376	A	V
FUC	fusA	PROTEIN	655	A	P
FUC	fusA	PROTEIN	463	D	G
FUC	fusA	PROTEIN	444	E	V
FUC	fusA	PROTEIN	468	E	V
FUC	fusA	PROTEIN	90	V	I
FUC	fusA	PROTEIN	114	P	H
FUC	fusA	PROTEIN	115	Q	L
FUC	fusA	PROTEIN	385	T	N
FUC	fusA	PROTEIN	404	P	L
FUC	fusA	PROTEIN	404	P	Q
FUC	fusA	PROTEIN	406	P	L
FUC	fusA	PROTEIN	434	D	N
FUC	fusA	PROTEIN	436	T	I
FUC	fusA	PROTEIN	438	H	N
FUC	fusA	PROTEIN	444	E	K
FUC	fusA	PROTEIN	451	G	V
FUC	fusA	PROTEIN	452	G	C
FUC	fusA	PROTEIN	452	G	S
FUC	fusA	PROTEIN	453	M	I
FUC	fusA	PROTEIN	457	H	Q
FUC	fusA	PROTEIN	457	H	Y
FUC	fusA	PROTEIN	461	L	K
FUC	fusA	PROTEIN	461	L	S
FUC	fusA	PROTEIN	464	R	C
FUC	fusA	PROTEIN	464	R	S
FUC	fusA	PROTEIN	464	R	H
FUC	fusA	PROTEIN	478	P	S
FUC	fusA	PROTEIN	556	G	S
FUC	fusA	PROTEIN	617	G	D
FUC	fusA	PROTEIN	651	M	I
FUC	fusA	PROTEIN	655	A	E
FUC	fusA	PROTEIN	656	T	K
FUC	fusA	PROTEIN	659	R	C
FUC	fusA	PROTEIN	659	R	H
FUC	fusA	PROTEIN	659	R	L
FUC	fusA	PROTEIN	659	R	S
FUC	fusA	PROTEIN	664	G	S
RIF	rpoB	PROTEIN	470	M	T

RIF	rpoB	PROTEIN	471	D	G
RIF	rpoB	PROTEIN	463	S	P
RIF	rpoB	PROTEIN	464	S	P
RIF	rpoB	PROTEIN	468	Q	K
RIF	rpoB	PROTEIN	468	Q	L
RIF	rpoB	PROTEIN	468	Q	R
RIF	rpoB	PROTEIN	471	D	Y
RIF	rpoB	PROTEIN	474	N	K
RIF	rpoB	PROTEIN	475		G
RIF	rpoB	PROTEIN	475		H
RIF	rpoB	PROTEIN	477	A	D
RIF	rpoB	PROTEIN	477	A	V
RIF	rpoB	PROTEIN	481	H	D
RIF	rpoB	PROTEIN	481	H	N
RIF	rpoB	PROTEIN	481	H	Y
RIF	rpoB	PROTEIN	484	R	H
RIF	rpoB	PROTEIN	486	S	L
RIF	rpoB	PROTEIN	527	I	F
RIF	rpoB	PROTEIN	550	D	G
CIP	gyrA	PROTEIN	84	S	A
CIP	gyrA	PROTEIN	84	S	L
CIP	gyrA	PROTEIN	85	S	P
CIP	gyrA	PROTEIN	88	E	K
CIP	grlA	PROTEIN	80	S	F
CIP	grlA	PROTEIN	80	S	Y

Supplementary Table 12 *S. aureus* chromosomal mutations from *Mykrobe predictor* resistance panel

Gene conferring resistance	Drug
blaZ	penicillin
mecA	methicillin
fusB	fusidic acid
fusC	fusidic acid
ermA	erythromycin<br>clindamycin (inducible)
ermB	erythromycin<br>clindamycin (inducible)
ermC	erythromycin<br>clindamycin (inducible)
ermT	erythromycin<br>clindamycin (inducible)
msrA	erythromycin
dfrA	trimethoprim

dfrG	trimethoprim
VGA(A)LC	clindamycin
tetK	tetracycline
tetL	tetracycline
tetM	tetracycline
aacA-aphD	gentamicin
vanA	vancomycin
mupA	mupirocin
mupB	Mupirocin

Supplementary Table 13 *S. aureus* genes from *Mykrobe predictor* resistance panel

Gene	Accession IDs
aacA-aphD	gi|452753789:36923-38362
blaZ	gi|408438460:3998-4885, NC_003140.1, NC_013331.1, NC_013337.1, NC_013351.1, NC_013352.1, NC_018972.1, NC_019008.1, NC_019010.1, NC_005054.1, NC_005011.1, NC_005127.1, NC_005951.1, NC_002952.2, NC_007168.1, NC_007931.1, NC_010066.1, NC_010063.1, NC_013550.1, NC_013653.1
dfrA	gi|46551:243-728, NC_005054.1, NC_007168.1, NC_012121.1, NC_012547.1, NC_014369.1
dfrB	NC_017347.1 , NC_017353.1, NC_017763.1, NC_017331.1, NC_017349.1, NC_018608.1, NC_022442.1, NC_022443.1, NC_022604.1, NC_002952.2, NC_007622.1, NC_010079.1
dfrG	gi|71040520:1013-1510, NC_017331.1, NC_022604.1
dfrK	FN812951.1, gi|308071690:7508-7999, gi|296274672:2919-3410, gi|325106579:1864-2355
ermA	gi|29165615:1685864-1686595, NC_002758.2, NC_002745.2, NC_017341.1, NC_017568.1, NC_017340.1, NC_020566.1, NC_020568.1, NC_009782.1, gi|690516876:92225-93106 gi|389870171:c47170-46433 gi|18542251|gb|AF466412.1| gi|18542253|gb|AF466413.1|

	gi|674653132|gb|KM194593.1
ermB	AB699882.1 (4971..5708), gi|402478084:4971-5708, NC_019213.1, NC_013963.1, NC_014475.1
ermC	gi|87159847:7865-8599, NC_001395.1, NC_019139.1, NC_020535.1, NC_006871.1, NC_007170.1, NC_007792.1
ermT	gi|288856430:917-1651,<br>HF583292 (11344..12078)
ermY	AB014481.1
fusA	NC_003923.1, NC_004461.1, NC_002758.2, NC_002745.2, NC_017568.1, NC_021670.1, NC_002952.2, NC_002953.3, NC_002951.2, NC_002976.3, NC_007168.1, NC_007350.1, NC_007622.1, NC_007793.1, NC_007795.1, NC_009487.1, NC_009632.1, NC_009641.1, NC_012121.1, NC_013450.1
fusB	gi|385782932:1336-1977, NC_017350.1
fusC	gi|49243355:52820-53458
mecA	gi|49482253:44919-46925, NC_003923.1, NC_002758.2, NC_002745.2, NC_017341.1, NC_017351.1, NC_017763.1, NC_017331.1, NC_017340.1, NC_017349.1, NC_018608.1, NC_002952.2, NC_002951.2, NC_002976.3, NC_007168.1, NC_007793.1, NC_009782.1, NC_010079.1
mecC	GI:871340505
msrA	gb|AF167161.1|:4100-5566, NC_022598.1
mupA	gb|DQ102365.1|:3302-6378
mupB	gb|JQ231224.1|:91-3192
tetK	gi|77102894:1138-2517, NC_019148.1, NC_006871.1
tetL	i|295980134:5846-7225
tetM	gb|M21136.1|STATETM:458-2377, NC_002758.2, NC_017341.1, NC_017331.1, NC_018608.1, NC_022604.1, NC_009782.1
vanA	gb|AE017171.1|:34299-35330, NC_019213.1, NC_014475.1

vanB	NC_005054.1 (34299 to 35330)
vanC	AF162694.1 (1411 to 2442)
VGA(A)LC	gi|111608743|gb|DQ823382.1

Supplementary Table 14: Accession codes of *S. aureus* gene alleles used to build target graph

drug	gene	alphabet	pos	ref	alt
rifampicin	*rpoB*	PROTEIN	425	F	*
rifampicin	*rpoB*	PROTEIN	426	G	*
rifampicin	*rpoB*	PROTEIN	427	T	*
rifampicin	*rpoB*	PROTEIN	428	S	*
rifampicin	*rpoB*	PROTEIN	429	Q	*
rifampicin	*rpoB*	PROTEIN	430	L	*
rifampicin	*rpoB*	PROTEIN	431	S	*
rifampicin	*rpoB*	PROTEIN	432	Q	*
rifampicin	*rpoB*	PROTEIN	433	F	*
rifampicin	*rpoB*	PROTEIN	434	M	*
rifampicin	*rpoB*	PROTEIN	435	D	*
rifampicin	*rpoB*	PROTEIN	436	Q	*
rifampicin	*rpoB*	PROTEIN	437	N	*
rifampicin	*rpoB*	PROTEIN	438	N	*
rifampicin	*rpoB*	PROTEIN	439	P	*
rifampicin	*rpoB*	PROTEIN	440	L	*
rifampicin	*rpoB*	PROTEIN	441	S	*
rifampicin	*rpoB*	PROTEIN	442	G	*
rifampicin	*rpoB*	PROTEIN	443	L	*
rifampicin	*rpoB*	PROTEIN	444	T	*
rifampicin	*rpoB*	PROTEIN	445	H	*
rifampicin	*rpoB*	PROTEIN	446	K	*
rifampicin	*rpoB*	PROTEIN	447	R	*
rifampicin	*rpoB*	PROTEIN	448	R	*
rifampicin	*rpoB*	PROTEIN	450	S	*
rifampicin	*rpoB*	PROTEIN	451	A	*
rifampicin	*rpoB*	PROTEIN	452	L	*
isoniazid	*katG*	PROTEIN	315	S	*
isoniazid	*fabG1*	DNA	-8	T	*
isoniazid	*fabG1*	DNA	-15	C	*
isoniazid	*fabG1*	DNA	-16	A	*
isoniazid	*fabG1*	DNA	-17	G	*
quinolones	*gyrA*	PROTEIN	85	H	*

quinolones	*gyrA*	PROTEIN	86	P	*
quinolones	*gyrA*	PROTEIN	87	H	*
quinolones	*gyrA*	PROTEIN	88	G	*
quinolones	*gyrA*	PROTEIN	89	D	*
quinolones	*gyrA*	PROTEIN	90	A	*
quinolones	*gyrA*	PROTEIN	91	S	*
quinolones	*gyrA*	PROTEIN	92	I	*
quinolones	*gyrA*	PROTEIN	93	Y	*
quinolones	*gyrA*	PROTEIN	94	D	*
kanamycin	*rrs*	DNA	1401	A	*
kanamycin	*rrs*	DNA	1402	C	*
kanamycin	*rrs*	DNA	1484	G	*
capreomycin	*rrs*	DNA	1401	A	*
capreomycin	*rrs*	DNA	1402	C	*
capreomycin	*rrs*	DNA	1484	G	*
amikacin	*rrs*	DNA	1401	A	*
amikacin	*rrs*	DNA	1402	C	*
amikacin	*rrs*	DNA	1484	G	*
ethambutol	*embB*	PROTEIN	306	M	*
kanamycin	*eis*	DNA	-10	C	T
ethambutol	*embB*	PROTEIN	406	G	D
ethambutol	*embB*	PROTEIN	406	G	S
streptomycin	*rpsL*	PROTEIN	43	K	R
streptomycin	*rpsL*	PROTEIN	88	K	R
streptomycin	*rrs*	DNA	513	C	*
streptomycin	*rrs*	DNA	514	A	*
streptomycin	*rrs*	DNA	515	G	*
streptomycin	*rrs*	DNA	516	C	*
streptomycin	*rrs*	DNA	517	C	*

Supplementary Table 15 *Mykrobe predictor* resistance panel for *M. tuberculosis*. Position is in nucleotides/amino-acids if alphabet is given as DNA/protein respectively. If an asterisk “*” is given in the final column, then any change from the reference base/amino acid is considered to cause resistance.
